# Evolution of the axial skeleton of early dinosaurs and the role of meristic and homeotic processes

**DOI:** 10.1098/rsos.261325

**Published:** 2026-10-14

**Authors:** André O. Fonseca, Iain James Reid, Maurício Silva Garcia, Rodrigo Temp Müller

**Affiliations:** ^1^ CAPPA, Universidade Federal de Santa Maria, Santa Maria, Brazil; ^2^ Programa de Pós-Graduação em Biodiversidade Animal, Universidade Federal de Santa Maria, Santa Maria, Brazil; ^3^ School of Engineering, The University of British Columbia, Kelowna, Canada

**Keywords:** Triassic, vertebral column, cervicalization, sacralization, Saurischia, Ornithischia

## Abstract

Changes in the axial skeleton played an important role in dinosaur evolution, with some of the oldest dinosaurs showing evidence of sacralization events. The increased number of early dinosaur discoveries over the last decade provides an opportunity to better characterize this phenomenon. It has led us to revise our understanding of axial evolution in early dinosaurs. Results support that the last common ancestor of Dinosauria would probably present 9 cervical, 16 dorsal and 2 sacral vertebrae (primordials). Early saurischians maintain a consistent number of vertebrae with presacral origin, and there is no evidence of meristic changes in these dinosaurs. A more complex scenario is observed in early ornithischians (including ‘silesaurids’). An insertion between the primordials occurs early in ornithischian evolution, and it appears to be maintained in ‘traditional’ ornithischians (Saphornithischia). Unambiguous evidence supports loss and gain of presacral vertebrae in ornithischians. Inserted sacrals across all dinosaurs are associated with a loss of a corresponding dorsal vertebra, supporting that these represent trunk vertebrae moved into the sacrum rather than novel vertebral unities. Changes in the axial skeleton of early dinosaurs are dominated by homeotic processes, with meristic processes restricted to derived members.

## 1. Introduction

The axial column represents the core of the skeleton, the structural axis to which both cranial and appendicular skeletons attach. It is responsible for enabling much of an animal's range of motion. These structural functions can be correlated with the evolution and ecology of a lineage (e.g. [1–4]). The axial skeleton in reptiles is typically divided into cervical, dorsal, sacral and caudal regions, although the boundary between cervical and dorsal regions can sometimes be difficult to distinguish [3,5]. In accordance with the ecology of a given clade, the morphology and number of vertebral elements in each axial zone may be modified (e.g. [3]). Therefore, one would expect anatomical disparity and diversity of specialization within the axial region to be greater in highly diverse lineages. Modifications to the number of vertebrae in each zonation can occur through two main pathways: meristic processes, in which the number of vertebral unities is altered, and homeotic processes, in which the axial zonation of the vertebra is changed [3,6,7]. The first process consists of the generation of new vertebrae (somitogenesis) or the loss of previously present ones, thereby changing the total number of vertebrae in the axial skeleton. The second process consists of the incorporation of vertebrae from one axial region into another; common examples would be trunk vertebrae being incorporated into the neck (cervicalization) or trunk and tail vertebrae being incorporated into the sacrum (sacralization). These homeotic processes occur through changes in Hox gene expression, in which trait characteristics of one axial zone (i.e. cervical) are expressed in vertebrae previously belonging to other regions (i.e. dorsal; e.g. [3,6,8–13]).

Once a vertebra is incorporated into another axial region, it is treated as belonging to the new region (e.g. a dorsosacral vertebra is considered a sacral rather than dorsal vertebra), as the incorporated vertebrae are functionally equal to the vertebrae previously present in the series. The fragmentary nature of the fossil record often hinders the identification of whether new vertebrae originate from somitogenesis or an incorporation process, therefore favouring the functional/anatomical criteria to identify which region a vertebra belongs to. Although it might be hard to identify when the process of somitogenesis and incorporation occurred, these transformations can be informative and contribute to our understanding of tetrapod evolution.

Dinosauria is a highly diverse clade of reptiles, containing the largest terrestrial animals ever known (sauropods) and some of the smallest vertebrates (e.g. hummingbirds). During the Mesozoic Era, dinosaurs diversified and tended to dominate terrestrial megafaunal niches in the Jurassic and Cretaceous periods. This diversity is reflected in their axial anatomy, which was transformed in different ways by different dinosaurian lineages. Among the examples that can be highlighted are the sacrum/sacral rods of ankylosaurians and euceratopsians that included up to 10 vertebrae fused into the synsacrum (e.g. *Edmontonia*, *Gargoyleosaurus*, *Protoceratops* [14,15]), and the highly elongated neck of sauropod dinosaurs at times containing more than 15 cervical vertebrae (e.g. *Euhelopus*, *Xinjiangtitan* [16,17]). Although not as remarkable as these extreme examples, modifications of the axial anatomy are characteristic of all dinosaurian lineages. A convergent increase in sacral complexity is observed in ornithischians, sauropodomorphs and theropods, with all lineages adding multiple vertebrae to their sacra (e.g. [18–21]). Nevertheless, the addition of vertebrae to the sacrum was one of the original defining characteristics of Dinosauria [22]. Although less widespread than the addition of sacral vertebrae, an increase in cervical count occurred multiple times in sauropodomorphs (e.g. *Plateosaurus*, *Xinjiangtitan* [17,23]), but was also seen in some ornithischians and theropods (e.g. *Coelophysis*, *Talenkauen* [24,25]). All the anatomical disparities observed in these derived dinosaurs stem from the more generalized condition observed in the earliest dinosaurs (e.g. [26,27]). Compared to derived forms, the first dinosaurs had a more conserved axial skeleton, with their vertebral count similar to the plesiomorphic condition of stem-archosaurs. Characters of the axial skeleton, and especially the number and origins of sacral vertebrae, have been considered significant to our understanding of dinosaur evolution (e.g. [18,20,27–30). In light of this importance, here, we conduct a revision of the axial evolution of early dinosaurs, aiming to understand the main drivers of axial evolution in these dinosaurs, the nature of these processes (meristic versus homeotic) and their importance for our understanding of dinosaur evolution.

## 2. Material and methods

### 2.1. Definition of scope

This study exclusively focuses on evaluating the evolution of the precaudal axial series. We opt not to evaluate the evolution of the caudal series as done in similar studies (e.g. [3]), owing to the rarity of complete series. Nevertheless, cases in which a caudal vertebra is incorporated into the sacrum will be taken into account in our analyses.

In the context of this study, we consider early diverging dinosaurs to be: non-eurypodan and non-pyrodontian ornithischians, herrerasaurians, non-sauropod sauropodomorphs and non-averostran theropods. This scope is chosen as it covers the earliest branching members of the three main dinosaurian lineages, and the majority of Triassic and Early Jurassic forms.

### 2.2. Terminology

Vertebrae incorporated into a new axial region are treated as belonging to their ‘recipient’ rather than their ‘donor’ regions. Therefore, cervical vertebrae acquired from the dorsal series are considered cervicals, and dorsosacral, inserted and caudosacral vertebrae are counted as sacrals.

The term ‘presacral count’ is used to identify the sum of the cervical and dorsal vertebrae. Here, we introduce the term ‘total presacral count’ to cover all vertebrae which would have their origins from presacral elements. The main difference between these terms is that the former considers only vertebrae that strictly belong to the cervical and dorsal series, whereas the latter also includes sacral vertebrae incorporated from the dorsal series, such as dorsosacral and inserted vertebrae. This distinction is important because accounting for sacralized vertebrae facilitates the detection of meristic processes, thereby providing a more accurate assessment of axial evolution. For example, *Dilophosaurus* has a presacral count of 24 vertebrae (cervical + dorsal), whereas it has a total presacral count of 25 vertebrae (cervical + dorsal + dorsosacral + inserted, although the last are absent in this taxon).

Whereas terms like ‘primordial sacral’, ‘dorsosacral’ and ‘caudosacral’ are widely used in reptile anatomy, the term ‘inserted sacral’ is less commonly employed. This vertebral condition was first recognized by Nesbitt [20], who proposed that one or more sacral vertebrae can be positioned between the primordial sacrals. We follow this definition, considering any vertebrae in between the primordials to represent an insertion/inserted sacral.

Although all ribs associated with sacral vertebrae may be considered ‘sacral ribs’, some sacral vertebrae, such as the primordial sacrals or insertions, possess a distinct anatomy that we refer to as ‘true sacral ribs’. In these vertebrae, the parapophysis has migrated ventrally onto the centrum and is either vertically aligned or nearly vertically aligned with the diapophysis [31]. Vertebrae bearing true sacral ribs can therefore be regarded as fully sacralized, displaying the characteristic rib of a sacral vertebra. On the other hand, vertebrae typically identified as dorsosacrals and caudosacrals lack this migration of the parapophysis [31], distinguishing them from fully sacralized vertebrae. These true sacral ribs have a one-to-one correspondence with their respective vertebral centra, as in the case of non-sacral ribs. Consequently, when a true sacral rib straddles the centra of successive vertebrae, it is possible to determine whether it belongs to the anterior or posterior vertebra. We emphasize that the term ‘true sacral rib’ should not be confused with terms such as ‘true sacrals’ or ‘proper sacral rib’ used in previous studies, as these refer to different concepts. For instance, in studies of ornithischians, ‘true sacrals’ denotes the vertebrae that bear individual sacral ribs [32–34].

### 2.3. Data collection

We revised available literature to compile a dataset containing the axial counts of early dinosaurs. When possible, specimens were observed first-hand. Some species have more than one interpretation of their axial counts, and others are reinterpreted here. Justification for these is presented in §3.1.

### 2.4. Evolutionary relationships

The early pan-avian phylogenetic tree follows the results of Müller & Garcia [35] at their most recent iterations [36,37]. Saphornithischian relationships follow Fonseca *et al.* [38]. Theropod relationships follow the ‘*Tawa* matrix’ in its most recent iterations [36,39,40], with *Notatesseraeraptor* positioned based on Zahner & Brinkmann [41]. Sauropodomorph relationships follow Fonseca *et al.* [42].

## 3. Results

A total of 33 early dinosaur taxa were deemed sufficiently preserved to be informative; their precaudal counts are summarized in table 1. Among these, ornithischians are represented by 8 species, herrerasaurians by 3 species, sauropodomorphs by 15 species and theropods by 8 species (figure 1).

**Table 1.** Axial counts of early-branching dinosaurs with informative axial skeletons.

sacrum									
taxa	cervical count	dorsal count	DSv	PS1	ISv	PS2	CSv	TPC	main reference
*Adeopapposaurus*	11	13	-	1	1	1	-	25	[43]
*Agilisaurus*	9	15	1/2	1	1/0	1	1	26	[44]
*Buriolestes*	9	16	-	1	-	1	1	25	[26]
*Coelophysis*	10	13	2	1	-	1	1	25	first-hand examination (IJR)
*Dilophosaurus*	9/10	15/14	1	1	-	1	1	25	[45] (amended)
*Efraasia*	10	14	1	1	-	1	-	25	[46]
*Eoraptor*	9	15/16	1/0	1	-	1	-	25	[47]
*Gnathovorax*	9	16	-	1	-	1	-	25	[48]
*Heterodontosaurus*	9	12	2/3	1	1/0	1	-	24	[34,49]
*Hexinlusaurus*	9	15	1/2	1	1/0	1	1	26	[50]
*Ignavusaurus*	?	15	1	1	-	1	-	?	[51]
*Lesothosaurus*	9	15	1/2	1	1/0	1	1	26	[52]
*Lewisuchus*	9	?	-	1	-	1	-	?	[53]
*Lucianovenator*	10	?	2	1	-	1	1	?	[54]
*Lufengosaurus*	10	14	1	1	-	1	-	25	[55]
*Macrocollum*	9	16	-	1	-	1	1	25	[56]
*Massospondylus*	10	14	-	1	1	1	-	25	[20,57]
*Minimocursor*	?	15	1/2	1	1/0	1	1	?	[58]
*Ngwevu*	10	14	-	1	?	?	?	?	[59]
*Notatesseraeraptor*	?	13/14	2/1	1	-	1	1	?	[41]
*Panguraptor*	10	13	?	?	?	?	?	?	[60]
*Plateosaurus*	10	15	-	1	-	1	1	25	[23]
*Sanjuansaurus*	9	?	-	1	-	1	-	?	[61] (amended)
*Sarahsaurus*	10	14	-	1	1	1	-	25	[62]
*Saturnalia*	9	15/16	1/0	1	-	1	0/1	25	[30,63]
*Scelidosaurus*	8	15	1/2	1	1/0	1	1	25	[64]
*Silesaurus*	9	15	-	1	1	1	-	25	[65] (amended)
*Staurikosaurus*	?	16	-	1	-	1	-	?	[66]
*Xingxiulong*	10	14	1	1	-	1	1	25	[67]
*Yizhousaurus*	10	14	1	1	-	1	-	25	[68]
‘*Sinosaurus*’ *sinensis*	9	15	1	1	-	1	1	25	[69]
‘*Syntarsus*’ *kayentakatae*	10	?	2	1	-	1	1	?	[70]
‘*Yunnanosaurus*’ *huangi*	10	14	1	1	-	1	-	25	[71]

*Note:* CSv, caudosacral vertebra; DSv, dorsosacral vertebra; ISv, inserted sacral; PS1, primordial sacral 1; PS2, primordial sacral 2; TPC, total presacral count; -, absent; ?, uncertain.

**Figure 1.**
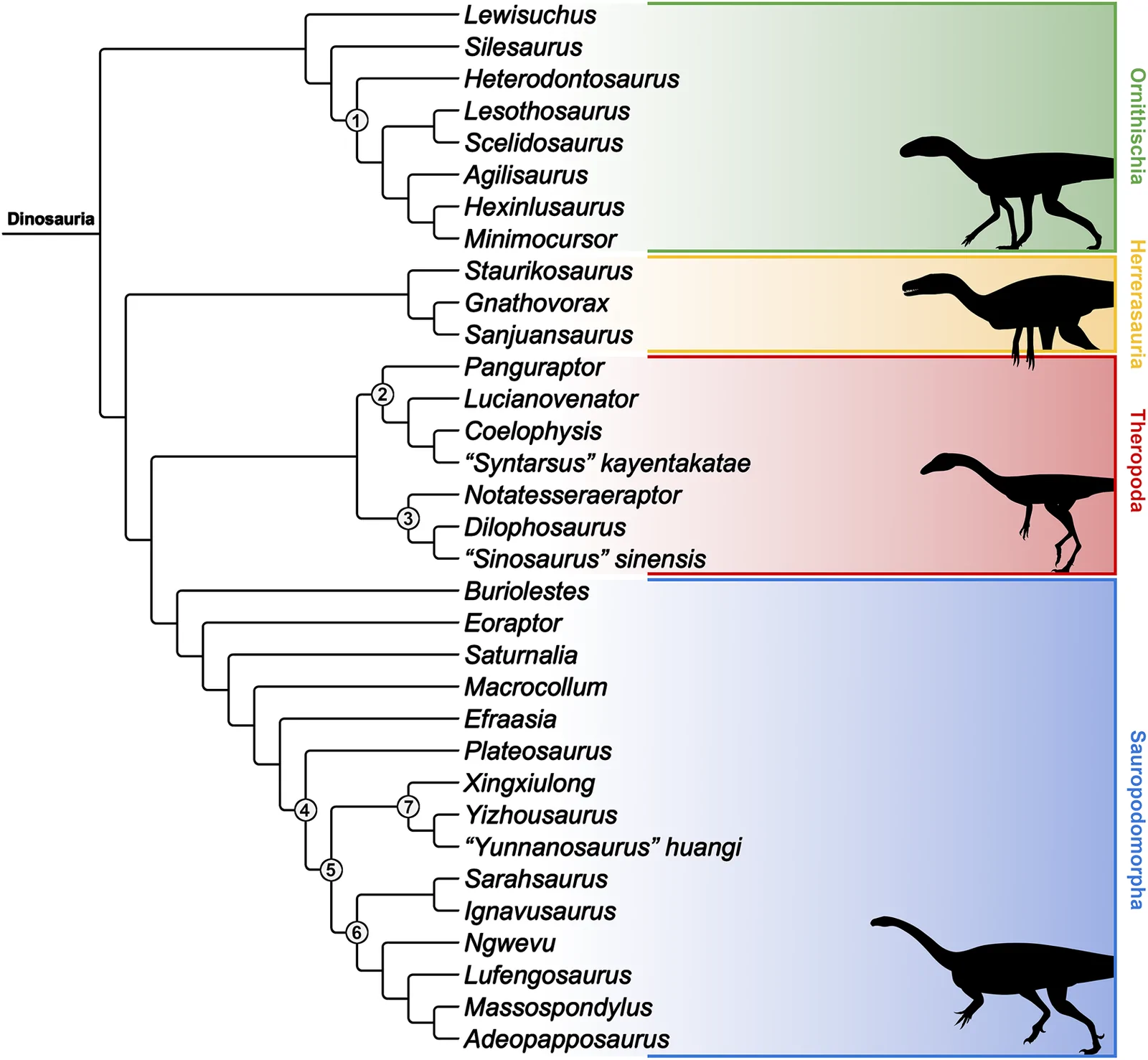
Phylogenetic relations of the early-branching dinosaurs evaluated in this study. Abbreviations: 1, saphornithischia; 2, coelophysoidea; 3, averostriformes; 4, plateosauria; 5, massopoda; 6, massospondylidae; 7, sauropodiformes.

Out of the 33 evaluated species, 23 taxa preserve the complete precaudal series (69.7%). From the remaining 10, 4 have an incomplete cervical series (12.1%), 4 have an incomplete dorsal series (12.1%) and 2 have incomplete sacral series (6.1%).

### 3.1. Disputed axial counts

Below, we discuss cases in which the number of elements is ambiguous or has been reinterpreted following our observations.

*Adeopapposaurus*: This taxon was described to have one dorsosacral in addition to a pair of primordial sacrals [43]. Owing to the similarities of its sacral ribs to those of *Massospondylus* and *Sarahsaurus*, we suggest it instead bears an inserted sacral vertebra (figure 2), rather than a dorsosacral.

**Figure 2.**
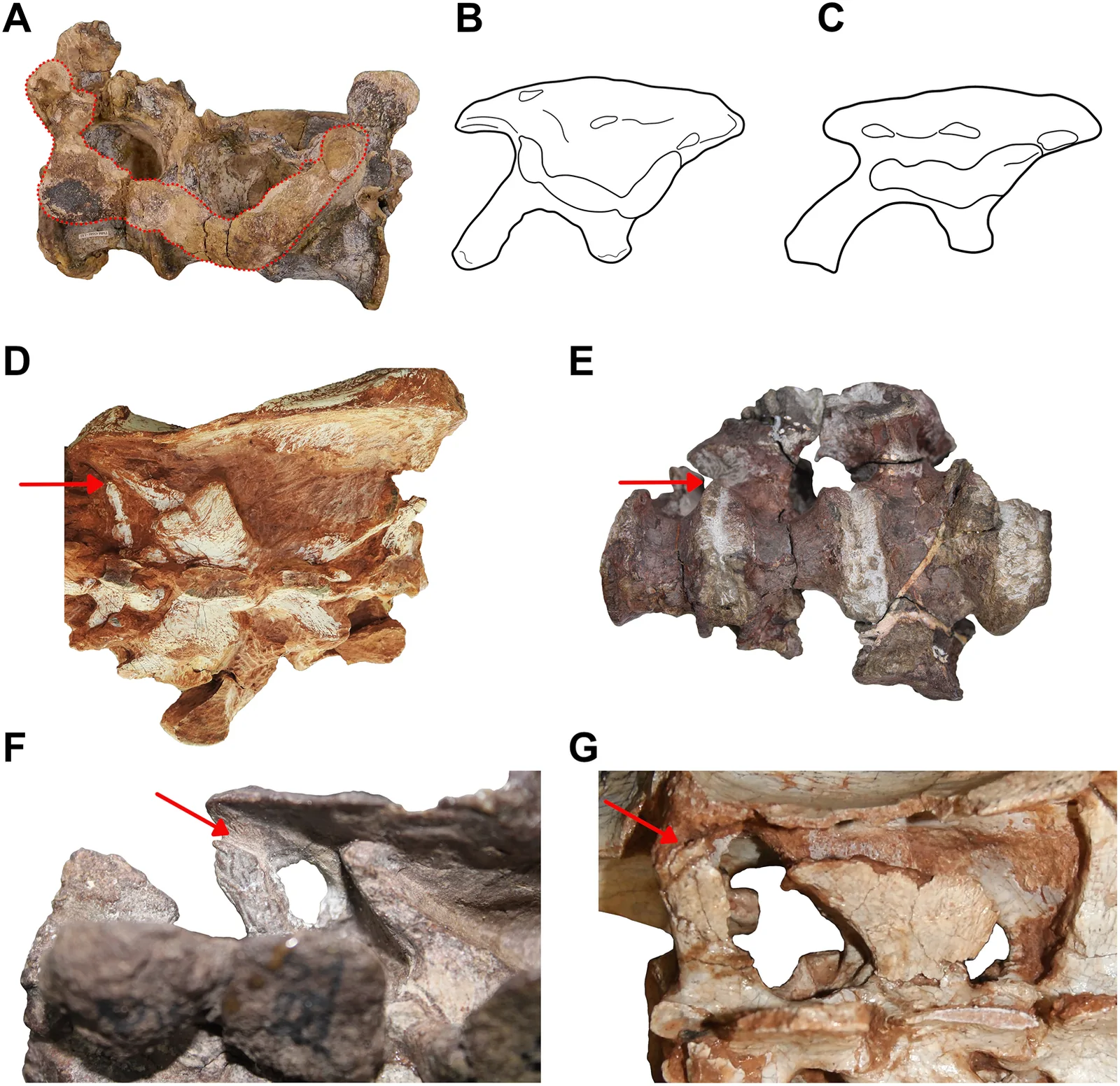
Selected axial elements of key early-branching dinosaurs. (A) Sacrum of *Sarahsaurus* (TMM 43646-2) in left lateral view, red dotted line highlighting the anatomy of the sacral ribs. (B) Schematic of the left ilium (mirrored) of *Adeopapposaurus* (PVSJ 569) in medial view (adapted from [43]). (C) Schematic of the right ilium of *Massospondylus* (BP/1/4934) in medial view (adapted from [57]). (D) Sacrum and ilia of *Gnathovorax* (CAPPA/UFSM 0009) in dorsal view, arrow highlighting the absence of contact between the transverse process of the last dorsal and the ilium. (E) Sacrum of *Sanjuansaurus* (PVSJ 605) in ventral view, arrow highlighting that the sacral rib is restricted to the first primordial sacral. (F) Sacrum and right ilium of *Eoraptor* (PVSJ 512) in dorsal view, red arrow highlighting the absence of contact between the transverse process and the ilium. (G) Sacrum and right ilium of *Saturnalia* (MCP-PV 3845) in dorsal view, arrow highlighting the contact between the transverse process and the ilium. None of the elements are set to scale.

*Anteavis*: This recently described taxon is proposed to bear a dorsosacral and a caudosacral vertebra, forming a four-vertebrae sacrum [39]. The putative incorporated vertebrae are not articulated to the ilia, nor have their neural arches been completely preserved. Their identification as sacrals is supported by a thickened posterior margin of the dorsosacral centrum, the shape of the caudosacral centrum and proposed articular facets in the medial surface of the ilia. Unfortunately, the pelvic girdle is preserved in articulation, and the entire surface of the ilia suffers from taphonomic weathering, hampering confirmation of the articular facets. Furthermore, the preserved bases of the transverse processes of the putative dorsosacral vertebra are dorsoventrally compressed (as in dorsal vertebrae not incorporated into the sacrum), instead of expanded/stout as in other saurischians with unequivocal dorsosacral vertebrae (figure 3), such as the sauropodomorph *Saturnalia* (MCP-3845-PV) and the neotheropods *Syntarsus kayentakatae* and *Dilophosaurus* [45]. The putative caudosacral centrum, however, is indeed morphologically different from typical caudal vertebrae, presenting dorsoventrally compressed articular facets and with the anterior articular facet larger than the posterior one. Furthermore, the dorsal margin of the caudosacral centrum appears to form a ‘lip’ in the anterior half of the centrum (figure 3D), suggesting the neural arch was positioned closer to the anterior margin of the centrum, differing it from unambiguous caudal vertebrae where it is more centrally placed. The sum of these features weakens the proposal that there is a dorsosacral vertebra in this taxon, although it strengthens the proposal for the presence of a caudosacral vertebra. After first-hand examination of the specimen, we follow the interpretation presented in Martínez *et al.* [39] for the caudosacral, but not for the dorsosacral vertebra.

**Figure 3.**
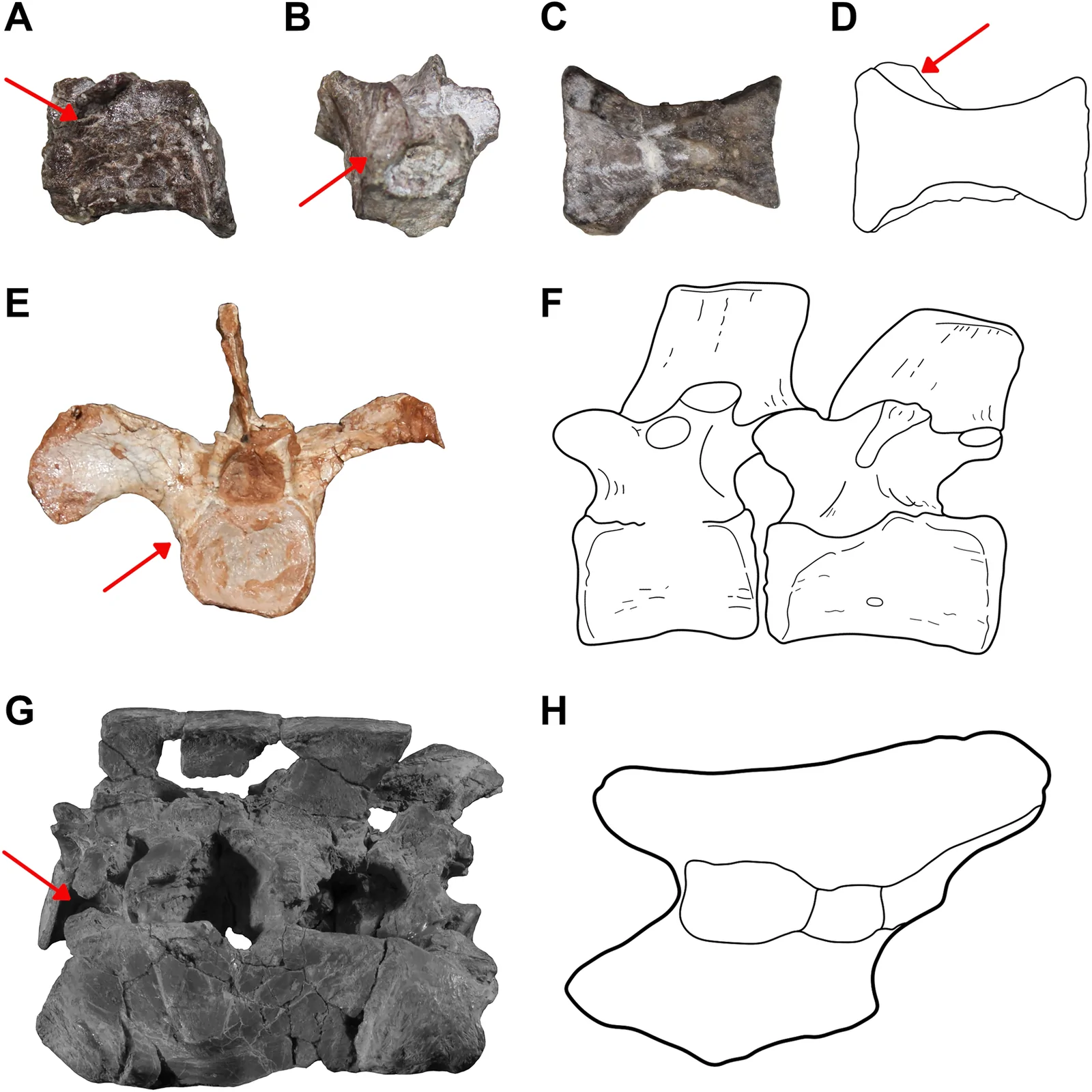
Selected axial elements of key early-branching dinosaurs. (A) Putative dorsosacral vertebra of *Anteavis* (PVSJ 1085) in left lateral view, arrow highlighting the transverse process without a dorsoventral expansion. (B) Putative dorsosacral vertebra of *Anteavis* (PVSJ 1085) in anterior view, arrow highlighting the transverse process without a dorsoventral expansion. (C) Caudal sacral vertebra of *Anteavis* (PVSJ 1085) in dorsal view. (D) Schematic of the caudosacral vertebra of *Anteavis* (PVSJ 1085) in ventral view (adapted from [39]), arrow highlighting the ‘lip’ formed by the dorsal margin of the centrum. (E) Dorsosacral vertebra of *Saturnalia* (MCP-PV 3845) in anterior view, arrow highlighting the dorsoventrally expanded transverse process. (F) Schematic of the last dorsal and proposed dorsosacral vertebra of *Scelidosaurus* (CAMSM X39256) in lateral view (adapted from [64]). (G) Sacrum and ilium of *Silesaurus* (ZPAL abIII/362) in lateral view (mirrored), iliac peduncles omitted, arrow highlighting the free transverse process of the last dorsal vertebra. (H) Ilium of *Silesaurus* (ZPAL AbIII/362) in medial view, showing the articular surfaces of the sacral ribs (adapted from [72]). None of the elements are set to scale.

*Coelophysis*: Though hundreds of specimens of *Coelophysis* are known, the common occurrence of articulated pelves and limited published description hinders the ability to compare interpretations of dorsosacral vertebrae. Colbert [24] identified five sacral vertebrae but did not describe the anatomy of the sacral ribs in lateral or ventral view where they could be compared to primordial sacrals and true sacral ribs. Spielmann *et al.* [73] described a sacrum referred to as *Coelophysis* (though not from the Ghost Ranch *Coelophysis* Quarry) that includes all five vertebrae and partial ribs, where it can be seen that a true sacral rib straddles the suture between sacrals 2 and 3. This rib can be identified as sacral rib 3, as sacral rib 4 straddles the suture between sacrals 3 and 4, and sacral 5 does not possess a true sacral rib, with the rib confined to the neural arch. Thus, while it appears that sacral 2 bears a true sacral rib and could be homologous with primordial sacral 1, this rib actually belongs to sacral 3, and sacral 2 cannot be homologous with primordial sacral 1 as proposed by Nesbitt [20]. The same anatomy can be seen in the disarticulated sacra preserved on the block TMP 1984.063.0001 (first-hand examination of TMP 1984.063.0037, TMP 1984.063.0038 and TMP 1984.063.0039), supporting the interpretation that the first two vertebrae in the sacrum of *Coelophysis* are dorsosacrals, with sacrals 3 and 4 corresponding to the primordial sacrals. The same anatomy is described for ‘*Syntarsus*’ *rhodesiensis*, where sacrals 1 and 2 lack true sacral ribs [74], as well as *Lucianovenator* [54], providing support to the shared sacral anatomy of coelophysoids and further reinforcing the identifications of sacral homology for *Coelophysis* presented here.

*Dilophosaurus*: The cervicodorsal junction in *Dilophosaurus* is uncertain owing to preservational limitations. Recently, it was proposed that *Dilophosaurus* possesses 14 cervical vertebrae, based on parapophyseal position, and only 11 dorsal vertebrae [45]. The authors noted that the identifications of vertebrae 11–14 were based on similarity to anterior presacrals rather than presumed location within the neck. Our re-evaluation of the vertebral column of *Dilophosaurus* shows that anatomical features associated with ribs participating in the pectoral girdle (i.e. anterior dorsal ribs) are found in presacral 11, and ambiguous in presacral 10. Consequently, *Dilophosaurus* is herein listed as having either 9 or 10 cervical and 14 or 15 dorsal vertebrae.

*Eodromaeus*: This species was described bearing 10 cervical vertebrae, 14 dorsal vertebrae and a sacrum composed of a dorsosacral and 2 primordial vertebrae [75]. However, the identification of the cervicodorsal junction in *Eodromaeus* was unspecified. After first-hand examination, we suggest that, based on the rib morphology, the identified cervical 10 could instead be the first dorsal vertebra. Available information is limited; therefore, cervical and dorsal counts are considered ambiguous for this reason. In the same fashion, the posterior dorsal vertebrae of *Eodromaeus* are embedded in matrix, making it currently impossible to determine whether a dorsosacral vertebra is present or not.

*Eoraptor*: The holotype was described as presenting 9 cervical, 15 dorsal and 3 sacral vertebrae, 1 of which was considered a dorsosacral [47,76]). However, the purported dorsosacral does not contact the ilium medially (figure 2F). It is possible that the contact occurred via an unpreserved sacral rib, as occurs in *Saturnalia* (figure 2G), and this vertebra would represent a dorsosacral. As is seen in *Saturnalia* (figure 3E), apart from the modified rib, the morphology of the dorsosacral is nearly identical to that of the dorsal vertebrae. Finally, this region in the holotype of *Eoraptor* is still partially embedded in matrix, hampering further observations. It is currently not possible to confirm the presence of the putative dorsosacral, and whether the taxon had 16 or 15 dorsal vertebrae.

*Herrerasaurus*: Information on axial configuration in *Herrerasaurus* is incomplete and comes from a composition of several specimens ([27]. This taxon has at least nine cervical vertebrae [77], which closely matches other early dinosaurs; thus, we agree with this count. Based on the dinosauromorph *Lagosuchus*, [27] assumed that the dorsal count in *Herrerasaurus* was 15, bringing the total presacral count to 24, 1 fewer than all other early dinosaurs with complete presacral series (e.g. *Buriolestes*, *Eoraptor*, *Gnathovorax*, *Macrocollum* [26,47,48,56,78]). Nonetheless, none of the *Herrerasaurus* specimens preserve a complete dorsal series, and the cervicodorsal transition is currently unknown in this taxon, rendering the total presacral count in *Herrerasaurus* ambiguous. For this reason, we do not include *Herrerasaurus* in our study; nevertheless, we offer an updated assessment of its axial anatomy. We assume this taxon would present 16 dorsal vertebrae based on closely related taxa (e.g. *Gnathovorax*, *Staurikosaurus*) with complete dorsal series, although this should be considered tentative. On the sacral vertebrae, some authors argue that *Herrerasaurus* presents a sacrum which includes a dorsosacral and two primordial sacral vertebrae [61,77]. However, there is no evidence that the supposed dorsosacral vertebra actually articulates medially with the ilia or that the transverse processes of said vertebra are modified compared with the preceding dorsal vertebra. Therefore, other authors suggested that the sacrum of *Herrerasaurus* contained solely the two primordial sacral vertebrae [27,66], as in other herrerasaurians (e.g. *Gnathovorax* and *Tawa* [48,78,79]). Herein, we follow the latter interpretation for this taxon.

*Lewisuchus*: There are two interpretations for the cervical count of this taxon: that it has seven cervical vertebrae [80], or that it has nine cervical vertebrae [53]. The hypothesis of seven cervical vertebrae was supported by a reduction in length of the cervical centrum and an increase in rib robustness from the seventh to the eighth presacral vertebrae in *Lewisuchus*, although the authors note that the parapophysis remains exclusively positioned on the centrum as far as the ninth presacral [80]. This justification is similar to one used in the study of *Silesaurus* [65], which will be discussed below. When describing a new specimen of *Lewisuchus*, [53] revised the interpretation of the cervical count in this taxon, in which a hypothesis of nine cervical vertebrae is supported. Whereas a transition in morphology between the seventh and eighth presacral vertebra in *Lewisuchus* is agreed upon, the authors argue that the parapophysis remaining exclusively in the centrum is a better landmark to determine the cervicodorsal transition [53]. We agree with the latter interpretation, with the position of the parapophysis being better evidence of this transition. Furthermore, no early pan-avian has a cervical count as low as seven vertebrae, which casts further doubt on it being the most adequate interpretation of the presacral series of *Lewisuchus*. Thus, we follow the conclusions of [53] and consider *Lewisuchus* to have nine cervical vertebrae.

*Massospondylus*: There is a dispute over whether this taxon has one dorsosacral or an inserted sacral [20,57]. Maintaining consistency with the homologous sacral morphology observed in *Sarahsaurus* and *Adeopapposaurus* (figure 2), we consider *Massospondylus* to have an inserted sacral.

*Notatesseraeraptor*: Whereas *Notatesseraeraptor* is described as preserving 13 dorsal and 4 sacral vertebrae [41], this interpretation is complicated by the disarticulation of the material and its incomplete preparation. Dorsal vertebrae 1–11 are preserved in articulation, and the identification of the first dorsal can be confirmed by the rib form and position underlying the anterior scapula [81]. Dorsal vertebra 11 is nearly completely hidden in the matrix, but remains articulated to dorsals 12 and 13 that can only be seen in CT images of the specimen. Hugi [81] identified four sacral centra and five sacral neural arches among the disarticulated vertebrae surrounding the ilia, with only sacrals 4 and 5, and the neural arches of sacrals 2 and 3, preserved in articulation. As only a fragmentary and disarticulated portion of the first sacral neural arch is preserved, it cannot be determined whether this vertebra articulated with the ilium and should be counted as a dorsosacral vertebra, or instead lay between the sacrum and dorsal series without articulation, in which case it would be considered the last dorsal vertebra. As there is no contradiction between preserved (albeit disarticulated) and expected numbers of dorsal–sacral vertebrae, we assume that the vertebral count as preserved is representative of the condition in life.

*Sanjuansaurus*: This species was described to have 9 cervical, 14 dorsal and 1 dorsosacral vertebrae [61]. The dorsal series was supposedly complete; however, the authors reported that at least three anterior dorsal vertebrae were partially or completely lost during preparation. The original interpretation of the dorsal number and sacral composition was based on *Herrerasaurus*, following the hypothesis that it would have 14 dorsal, 1 dorsosacral and 2 primordial sacral vertebrae [61]. The axial anatomy of herrerasaurids has been historically hard to interpret (see [66]), but became more clear with the description of *Gnathovorax*, which is currently the only herrerasaurid to present a complete and well-preserved precaudal series [48,78], with 16 dorsal vertebrae and 2 primordial sacral vertebrae. This well-preserved taxon allows us to reinterpret the axial anatomy of herrerasaurids. Based on comparisons between these taxa and first-hand observation, we suggest that *Sanjuansaurus* does not bear a dorsosacral, and that the putative element actually represents the last dorsal vertebra (figure 2E). However, even if the reported dorsosacral is considered the last dorsal vertebra, *Sanjuansaurus* would still present one fewer dorsal vertebra than *Gnathovorax*. Because the number of dorsal vertebrae present in the other herrerasaurids *Herrerasaurus* (see above) and *Staurikosaurus* (see below) is also ambiguous, we suggest *Sanjuansaurus* bears nine cervicals, an uncertain number of dorsals (between 15 and 16), and a sacrum exclusively composed of the two primordial sacrals.

*Scelidosaurus*: The sacral count of this taxon is unclear, with two possibilities suggested by Norman [64]. This taxon has either 16 dorsal vertebrae and 4 sacral vertebrae, or 15 dorsal vertebrae and 5 sacral vertebrae, with ‘dorsal’ 16 of the first hypothesis actually representing a dorsosacral in the second hypothesis. The morphology of this last vertebra is recognized as distinct from the remaining vertebrae, with a broad centrum, a flat posterior articular surface and the neural arch positioned further anteriorly [64]. Despite these similarities to sacral vertebrae, it lacks an individualized sacral rib [64]. This set of features suggests that this vertebra can represent a dorsosacral element (figure 3F). It is challenging to conclusively assess the morphology of this species, as no adult is known and ontogenetic factors might account for some of the unusual features of this vertebra in addition to differences between specimens [64]. Although future research might dispute it, we favour the hypothesis in which the sacrum of *Scelidosaurus* bears five sacral vertebrae. This hypothesis accounts for the unusual morphologies of the ‘last dorsal’ and resembles the sacral morphology observed in other earlier branching saphornithischians in having one dorsosacral, three ‘main’ sacral vertebrae and one caudosacral (see §4).

*Silesaurus*: The delineation of the axial zonations in this taxon has been a subject of debate. Originally, it was interpreted to have either 9 or 11 cervical vertebrae [82], although a subsequent contribution argued the presence of only 7 [65]. This distinct interpretation stems from a change in rib morphology and reduction in central length observed between the seventh and eighth presacral vertebrae (ZPAL AbIII-1930), leading the authors to consider this the boundary between the cervical and dorsal regions. However, it appears that the cervicodorsal transition in ZPAL AbIII-1930 is damaged, hampering this interpretation (see [53]), and other authors still consider it to bear more than seven cervical vertebrae (e.g. [53,83,84]. No other early dinosauromorph exhibits such a low cervical count, including the closely related *Lewisuchus*, casting further doubt on this assessment. We consider it most parsimonious to assume *Silesaurus* would bear nine cervical vertebrae, a condition shared with *Lewisuchus*, saphornithischians and the majority of early dinosauromorphs. Owing to the uncertainties in the cervical count, the number of dorsal vertebrae is also variable. According to Piechowski & Dzik [65], *Silesaurus* bears 16 dorsal vertebrae, with this number being reduced to 14 based on the amended interpretation of cervical count. The sacrum of *Silesaurus* was originally interpreted to bear four sacral vertebrae, although most now consider the putative first sacral to be a dorsal vertebra, as it does not articulate to the ilium (figure 3G) [20]. Despite the different interpretations of the cervicodorsal boundary, the presacral count should remain stable. In *Silesaurus*, the presacral series is composed of 23 vertebrae [65]; this number would be amended to 24 as the ‘first sacral’ is reinterpreted as the last dorsal.

*Staurikosaurus*: The number of trunk and sacral vertebrae is disputed in this taxon, as discussed by Novas *et al.* [66]. Its sacrum has been interpreted to bear one caudosacral (e.g. [85]) or exclusively the two primordial sacrals (e.g. [66,86]). Based on observation of *Gnathovorax*, the only herrerasaurid with a complete and well-preserved precaudal series [48,78], and the discussion presented in Novas *et al.* [66], we consider *Staurikosaurus* to have 16 dorsal vertebrae and a sacrum composed exclusively of the 2 primordial sacrals. The total number of cervical vertebrae, and therefore the total number of presacral vertebrae, is unknown in this taxon because it does not preserve the entire cervical series [85].

### 3.2. General trends

#### 3.2.1. Herrerasauria

The total presacral count consists of 25 vertebrae, shared with all early saurischians. There is no evidence of modifications to the cervical count, maintaining the plesiomorphic nine vertebrae. The same seems true for the dorsal series, with 16 vertebrae, although its record is less complete. Contrary to the other three dinosaurian radiations, all herrerasaurians retain a sacrum composed exclusively of the two primordial sacrals.

#### 3.2.2. Theropoda

The total presacral count of theropods appears to be stable as in other saurischians. All taxa identified bear 25 vertebrae with a presacral origin, either within the cervical, dorsal or dorsosacral regions. No insertions can be identified in the sampled taxa. Although Nesbitt [20] identified inserted sacral vertebrae in early branching (e.g. *Coelophysis* and *Dilophosaurus*) and derived theropods (e.g. *Allosaurus* and *Carnotaurus*), we disagree on the presence of an insertion on the first two taxa. The plesiomorphic cervical and dorsal counts are difficult to establish owing to the uncertainty in *Eodromaeus* and *Dilophosaurus* cervical counts, and the fluctuation in phylogenetic relationships of Coelophysoidea. It is possible that a cervical count of 10 is either plesiomorphic for Theropoda or convergent between Coelophysoidea and some Averostriformes (e.g. *Dilophosaurus*). The increase in cervical count corresponds to a decrease in dorsal count wherever observed, validating a homeotic shift in position rather than loss of a vertebra caused by changes in somitogenesis.

Sacral counts of four or five are typical for theropods, with five sacrals found in coelophysoids and derived averostrans (but ambiguous in *Notatesseraeraptor*; table 2). The early-branching theropod *Eodromaeus* is suggested to present three sacrals, with two primordial vertebrae and a putative dorsosacral, although first-hand examination reveals that the presence of a dorsosacral cannot be confirmed (see §3.1). More derived theropods incorporate a caudosacral vertebra, resulting in a sacrum composed at least of the two primordial vertebrae and one caudosacral, as observed in the recently described *Anteavis* [39]. By contrast, incorporation of a dorsosacral vertebra can only be confirmed in Neotheropoda, with a second dorsosacral vertebra with ambiguous phylogenetic distribution occurring in neotheropods. Taxa bearing an additional dorsosacral vertebra consistently show a compensatory reduction in the number of dorsal vertebrae. This pattern independently supports a conservative, stable count of presacral vertebrae despite sacral reorganization.

**Table 2.** Sacral counts of early-branching theropods with informative sacra.

sacrum						
taxa	DSv	PS1	ISv	PS2	CSv	main reference
*Anteavis*	?	1	-	1	1	first-hand examination (MSG & RTM)
*Coelophysis*	2	1	-	1	1	first-hand examination (IJR)
*Dilophosaurus*	1	1	-	1	1	[45]
*Eodromaeus*	?	1	-	1	-	first-hand examination (MSG & RTM)
*Lucianovenator*	2	1	-	1	1	[54]
*Megapnosaurus*	2	1	-	1	1	[74]
*Notatesseraeraptor*	2/1	1	-	1	1	[41]
*Pendraig*	2/1	1	0/1	1	?	[87]
‘*Sinosaurus*’ *sinensis*	1	1	-	1	1	[69]
‘*Syntarsus*’ *kayentakatae*	2	1	-	1	1	[70]
‘Shake n bake*’* theropod	2	1	-	1	1	[70]
‘Snyder’ theropod	2	1	-	1	1	[73]

*Note:* CSv, caudosacral vertebra; DSv, dorsosacral vertebra; ISv, inserted sacral; PS1, primordial sacral 1; PS2, primordial sacral 2; -, absent; ?, uncertain.

#### 3.2.3. Sauropodomorpha

The total presacral count of sauropodomorphs is stable, as observed in the remaining saurischians. Most, if not all, taxa bear 25 vertebrae which are presacral in origin, once dorsosacrals and insertions are accounted for. The possible exceptions, *Ngwevu* and taxa with inserted sacrals, are further explored in §4. The earliest branching sauropodomorphs present the plesiomorphic condition of nine cervical vertebrae (e.g. *Buriolestes*, *Eoraptor* and *Macrocollum*). All sauropodomorphs more derived than Unaysauridae have at least 10 cervicals, and the massospondylid *Adeoppaposaurus* has an 11th cervical. These additional cervical vertebrae are consistently correlated with the loss of a dorsal vertebra, indicating that they originate through cervicalization of dorsal elements.

All sauropodomorphs seem to possess a minimum of three sacral vertebrae; however, the identity and origin of the third element vary across the clade, usually represented by either a dorsosacral or caudosacral vertebra (see [42]). This variation persists up to Massopoda, where sacral composition becomes stabilized, with the third element consistently represented by a dorsosacral or an inserted vertebra. Within massopodans, a caudosacral is restricted to taxa bearing four or more sacral vertebrae and is never the third sacral element (e.g. *Xingxiulong*). Lack of well-preserved axial series in later branching sauropodiforms hampers our understanding of the distribution of a four-vertebrae sacrum. Near-sauropods (sensu [88]) can have sacra composed of five vertebrae, with the inclusion of one additional dorsosacral (e.g. NMQR 1551 [89,90]).

#### 3.2.4. Ornithischia

Ornithischians exhibit a greater degree of variability in total presacral count than saurischians, ranging from 23 to 26 depending on the taxon and on which hypothesis of sacral homology is adopted. Following the preferred interpretations of cervical count in ‘silesaurids’, most ornithischians sampled show a cervical count of nine vertebrae. The exception is *Scelidosaurus*, in which one cervical vertebra was probably lost. Variability in the presacral count mostly occurs in the dorsal series, especially with the reduced dorsal count in *Heterodontosaurus*. In the latter, although some dorsal vertebrae were incorporated into the sacral series, the total presacral count remains reduced relative to the plesiomorphic condition.

The sacrum of the earliest branching members is exclusively composed of the primordial sacrals (e.g. *Lewisuchus*), and one additional vertebra is inserted between the primordial sacrals in early sulcimentisaurians (e.g. *Gondwanax*, *Silesaurus* [19]). All saphornithischians exhibit a sacrum composed of five vertebrae, although their homology is not immediately clear. The first is considered a dorsosacral, based on the position of its transverse process and lacking an individualized sacral rib (sharing a rib with the second sacral), the fourth is considered the second primordial sacral based on previous attempts of identifying it and its consistent position in saurischians (see §4), and the fifth is considered a caudosacral based on its location posterior to the second primordial sacral and its transverse process articulating with either the postacetabular process or the base brevis shelf. In heterodontosaurids, this five-vertebra condition is further modified by addition of a second dorsosacral, resulting in a sacrum composed of six vertebrae. As previously argued, *Scelidosaurus* might bear only four sacral vertebrae, although this is not the hypothesis favoured here.

## 4. Discussion

### 4.1. Axial evolution across early saurischians

The presacral count remains roughly consistent across the archosaur stem, with species of many groups having around 24–26 presacral vertebrae (e.g. *Protorosaurus*, *Prolacerta*, *Azendohsaurus*, *Proterosuchus*, *Euparkeria*, *Chanaresuchus* [91,92], MD Ezcurra 2026, personal communication), although some proterochampsids show a reduced presacral count of 21 vertebrae (*Pseudochampsa* [93]). In these basal forms, the sacrum is nearly exclusively composed of the two primordial sacrals [20,83], with *Doswellia* representing an exception [94]. Presacral counts are difficult to determine in early-branching pan-avians. *Lagosuchus* and *Yarasuchus* represent the two best-preserved taxa, though neither has a complete series [95,96]. It can be confirmed that *Lagosuchus* bears at least 24 presacral vertebrae, but no individual preserves a complete presacral series (MD Ezcurra, personal communication). The description of *Yarasuchus* suggested that it bears 8 cervical and 17 dorsal vertebrae [95], but these vertebrae were not described in detail. Another challenge in assessing the axial count of *Yarasuchus* is that its remains were not preserved in articulation and are intermingled with those of an allokotosaurian, *Pamelaria dolichotrachela* [97]. Most, if not all, early-branching pan-avians bear only two sacral vertebrae (e.g. *Ixalerpeton*, *Lagerpeton*, *Lagosuchus*, *Mambachiton*, *Teleocrater*, *Yarasuchus* [95–101]. A possible exception is the enigmatic *Nyasasaurus*, which was described as presenting three sacral vertebrae: two primordial and one insertion [102]. Taken together, this pattern suggests that the presacral series and sacrum retained a largely consistent anatomical configuration along the archosaur stem and into early pan-avians.

Available evidence suggests that the common ancestor of saurischians had a presacral count of 25 vertebrae: 9 cervicals and 16 dorsals (figure 4). A sacrum composed of only the primordial sacrals is observed in most non-dinosaur archosauromorphs, earliest ornithischians and herrerasaurians, further supporting this is the ancestral condition in saurischians. Current evidence does not support modifications in the axial skeleton of herrerasaurians. Previously reported variability in dorsosacral and caudosacral counts is best explained by the absence of complete, well-preserved axial series, which has hindered reliable comparisons. It should be highlighted that all herrerasaurians with significantly complete and articulated axial series are members of Herrerasauridae, and that the axial skeleton of all non-herrerasaurid herrerasaurians is represented by isolated remains or corresponds to compositions of multiple individuals (e.g. *Chindesaurus*, *Tawa* [79,103]). Although their presacral series are incomplete, the sacra of *Chindesaurus* and *Tawa* confirm that they would bear only two sacral vertebrae [79,103], as in herrerasaurids, supporting a conservative sacral count in Herrerasauria. The recently described *Maleriraptor* might bear a caudosacral vertebra [36], although the authors also consider it to possibly represent the first caudal vertebra. The absence of well-preserved presacral series might suggest that there is the possibility of more variability within herrerasaurians, although the sacrum seems more conservative.

**Figure 4.**
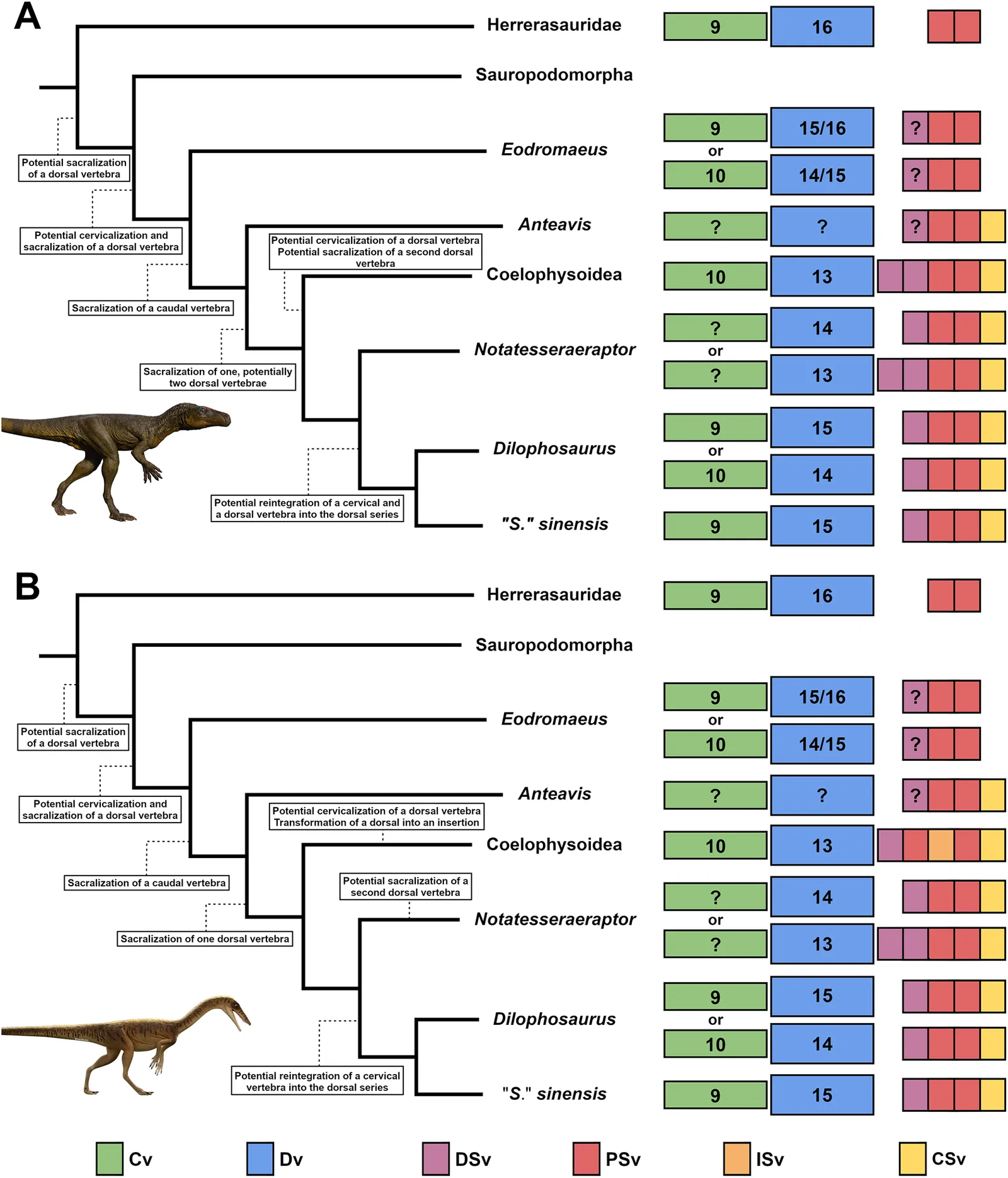
Evolution of the precaudal axial skeleton of early-branching non-sauropodomorph saurischians. (A) Scenario considering coelophysoids to bear two dorsosacral vertebrae. (B) Scenario considering coelophysoidea to bear one dorsosacral vertebra and one insertion. Artistic reconstruction by Caio Fantini (A) and Márcio L. Castro (B). Abbreviations: Cv, cervical vertebrae; CSv, caudosacral vertebra; Dv, dorsal vertebrae; DSv, dorsosacral vertebrae; ISv, inserted sacral vertebra; PSv, primordial sacral vertebrae.

Early-branching theropods display the plesiomorphic total presacral count of 25 vertebrae. There is evidence for at least one cervicalization event in the sample, and at most two. The number and timing of occurrence of these cervicalization events are dependent on the cervical count in *Eodromaeus*. Whereas coelophysoids clearly possess 10 cervicals, it is not certain whether non-coelophysoid theropods also possess 10 cervicals or whether the cervicalization is synapomorphic of that group. Cervical count in Averostra is also uncertain, with presacrals often remaining undifferentiated [104]. It is possible that only a single cervicalization event occurred ancestrally with migrations of one cervical back into the dorsal series in taxa that display nine cervical vertebrae, such as ‘*Sinosaurus*’ *sinensis*. The evaluation of cervicalization in more derived theropods is beyond the scope of this contribution.

Sacralization within theropods appears to be consistent, but the interpretations of vertebral origins here disagree with previous studies on the presence of an insertion sacral. All neotheropods possess at least one dorsosacral, though whether this sacralization is homologous with the same event in sauropodomorphs is reliant on the reconstructed ancestral state in the latter group (see below). A sacralization from the caudal series is shared across all neotheropods, bringing the minimum sacral count within the group to four. Whereas the presence of a dorsosacral across non-neotheropod theropods remains ambiguous, the recently described *Anteavis* confirms that the incorporation of a caudal vertebra into the sacrum occurred prior to the origin of Neotheropoda. Whereas some authors consider an insertion to be present in early-branching theropods based on the position of articulations with the ilium [20,87], only the penultimate and antepenultimate sacrals bear a true sacral rib where it can be observed. This supports the interpretation that the third sacral vertebra of taxa such as *Coelophysis* is the first primordial sacral, rather than the second vertebra as interpreted in the aforementioned studies. If our hypothesis on homology is incorrect and an insertion is present in coelophysoids, the implications for evolution of other theropods would be minimally affected, limited to a discussion if two dorsosacrals are present in *Notatesseraeraptor* and how it relates to the condition of other averostriformes (see figure 4B). Regardless of which pathway causes the acquisition of this fifth sacral vertebra, the added vertebra when present, as in coelophysoids and some averostrans (e.g. *Monolophosaurus* [105]), is associated with the loss of a dorsal element rather than a new vertebra being formed in the sacrum. Therefore, if coelophysoids bear an insertion, they would provide further evidence for our hypothesis that the inserted sacral derives from the dorsal series (see §4). In summary, independent of which hypothesis of coelophysoid sacral homology is correct, all theropods within our scope have a total presacral count of 25 vertebrae (cervicals + dorsals + dorsosacrals + insertions), matching the proposed plesiomorphic condition of Dinosauria.

All early-branching sauropodomorphs share the ancestral total presacral count of 25 vertebrae (figure 5), although there are possible exceptions. Primarily, taxa considered to have an inserted sacral (*Adeoppaposaurus*, *Massospondylus* and *Sarahsarus*) appear to be missing one presacral vertebra. However, this would be explained by the hypothesis that the inserted sacral originates from the dorsal series (see below), fulfilling the count of 25 vertebrae with a presacral origin. Another potential exception is seen in *Ngwevu*, as its holotype preserved a complete presacral series (24 vertebrae) and the first primordial sacral, suggesting a presacral count of only 24 vertebrae [59]. This could potentially suggest loss of a dorsal element in this taxon. However, *Ngwevu* is a massospondylid, as are the other three sauropodomorphs that present an inserted sacral vertebra. It is possible that this species might bear an inserted sacral vertebra, fitting the axial count observed in the remaining saurischians. The available fossils do not allow us to unambiguously confirm the total presacral count of *Ngwevu*; thus, we do not consider it sufficient evidence to contest the pattern of 25 total presacral count observed across all other early saurischians. During the remainder of §4, we will consider the total presacral count stable and unchanged in this group.

**Figure 5.**
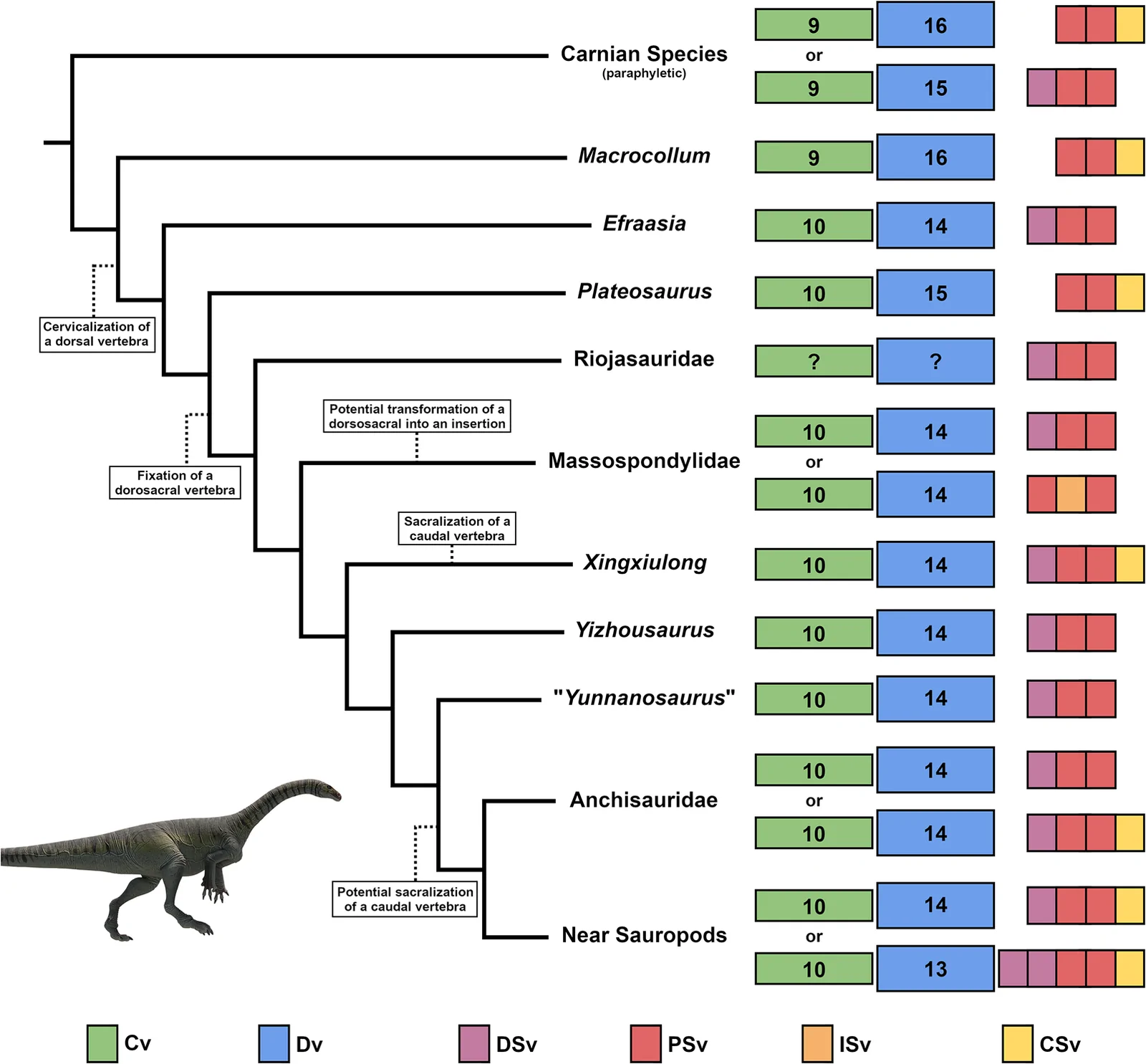
Evolution of the precaudal axial skeleton of early-branching sauropodomorphs. Artistic reconstruction by Márcio L. Castro. Abbreviations: Cv, cervical vertebrae; CSv, caudosacral vertebra; Dv, dorsal vertebrae; DSv, dorsosacral vertebrae; ISv, inserted sacral vertebra; PSv, primordial sacral vertebrae.

The earliest branching sauropodomorphs have the plesiomorphic count of nine cervical vertebrae, although two cervicalization events are identified in the sample. The first event occurs in a yet unnamed node containing the sauropodomorphs more derived than unaysaurids, in which the first dorsal is incorporated into the cervical series, now comprising 10 vertebrae [42]. The second event is autapomorphic to *Adeoppaposaurus*, a taxon which incorporated a second dorsal vertebra into its cervical series, totalling 11 vertebrae.

The sacralization process in sauropodomorphs represents a more complex scenario. As noted in previous studies (e.g. [42]), Carnian sauropodomorphs show cases of sacralization via incorporation of dorsal vertebrae (e.g. *Mbiresaurus* and possibly *Eoraptor* [47,106]), caudal vertebrae (e.g. *Buriolestes* [26,100]) or putative individual variation where both conditions are present in the same taxa (e.g. *Saturnalia* [30,63]). Despite being acquired by different pathways, all Carnian sauropodomorphs seem to bear at least three sacral vertebrae (although *Eoraptor* could be an exception). Post-Carnian taxa continue to exhibit this variation and enhance the disparity, with some taxa also presenting an inserted sacral vertebra (e.g. *Adeopapposaurus*, *Massospondylus* and *Sarahsaurus*). The large variability in sacral composition disappears in Massopoda, with all members having one dorsosacral (or insertion) and two primordial sacrals. A caudosacral reappears in more derived taxa, such as both species of *Xingxiulong*, some anchisaurids (e.g. *Leonerasaurus* and possibly *Mussaurus*) and species close to the origin of sauropoda (e.g. NMQR 3314), resulting in a four-vertebrae sacrum [21,67,107–109]. In addition, the sacrum of specimen NMQR 1551 bears five sacral vertebrae, incorporating one additional dorsosacral vertebra to the previous four-vertebrae condition [89,90].

The large variation in the sacral anatomy of early-branching sauropodomorphs prevents a more thorough understanding of their evolution. Despite this challenge, some patterns can be identified. Three sacral vertebrae are near universally present across sauropodomorphs (and all eusaurischians), and it seems reasonable to consider this to be their ancestral condition. Otherwise, the high plasticity observed in Carnian sauropodomorphs (dorsosacral vs. caudosacral vs. polymorphism) suggests that this number might have been achieved independently. Furthermore, if the ‘dorsosacral’ of *Eoraptor* does not touch its ilium, it would offer evidence of a sauropodomorph with only two sacral vertebrae. Pending more solid evidence, we still favour the first scenario as the most parsimonious, but highlight that the differences in which axial region is sacralized might suggest an alternative hypothesis. The high plasticity is maintained until the root of Massopoda. All massopodans bear a dorsosacral vertebra, and a caudosacral is only present if a fourth sacral is incorporated. Therefore, it is safe to consider that the ancestral massopodan would bear one dorsosacral and no caudosacral vertebra. Exceptions to this pattern are members of Massospondylidae that bear an inserted sacral (e.g. *Sarahsaurus*), although the inserted condition in these taxa is most probably a variation of the dorsosacral condition (see §4 on the origins of inserted sacrals). A new sacralization event, incorporating a caudal vertebra, occurs in different massopodan lineages: *Xingxiulong*, anchisaurids and near sauropods [21,67,107–109]. The position of *Xingxiulong* suggests that its caudosacral vertebra was acquired independently from anchisaurids and near sauropods. Unfortunately, the limited number of well-preserved axial series prevents testing whether the caudosacral vertebra of anchisaurids and near sauropods is homologous or convergently acquired, further complicated by one anchisaurid not bearing a caudosacral vertebra (e.g. *Anchisaurus/Ammosaurus* [110]). In conclusion, at least four independent cases of sacralization occur in Massopoda, with an uncertain number occurring in earlier branching members.

There is no substantial evidence for somitogenetic (meristic) changes in the precaudal axial series of early-branching saurischians. In all observed cases, the acquisition of an additional cervical, dorsosacral and inserted vertebra coincides with the loss of a dorsal element. This strongly suggests that the origin of these vertebrae occurs through changes in Hox gene expression (homeotic processes), probably causing some anterior dorsals to develop cervical-like anatomy and posterior dorsals to develop sacral-like anatomy. Our examination supports the conclusion that the earliest saurischians maintained a consistent total presacral count of 25 vertebrae (cervicals + dorsals + dorsosacrals/inserted elements), with no evidence for somitogenesis of either kind (vertebral gain or vertebral loss). By contrast, the developmental origin of caudosacral vertebrae cannot be robustly assessed owing to the rarity of fully preserved caudal series.

### 4.2. Axial evolution across early ornithischians

Assessing axial skeletal evolution in ornithischians is more challenging than in any other dinosaur clade. Despite growing evidence supporting ‘silesaurids’ as a paraphyletic series of early-branching ornithischians [35,38,111–113], this scenario is not unequivocal across all phylogenetic analyses (e.g. [66,114,115]). The discussion presented here is framed under the hypothesis that ‘silesaurids’ represent a grade of early ornithischians (figure 6A); however, the conclusions are not strongly contingent on this assumption. This is because the axial series of *Lewisuchus* and *Silesaurus* closely resemble those of early-branching saurischians, differing primarily in the presence of an inserted sacral vertebra in *Silesaurus*. Consequently, the inferred axial anatomy of the ornithischian ancestor would remain broadly similar regardless of the phylogenetic placement of ‘silesaurids’. Moreover, the discussion of axial evolution within saphornithischians is largely self-contained and would not be substantially altered if ‘silesaurids’ were recovered as paraphyletic relative to core ornithischians or as a dinosaurian outgroup. Still, below, we discuss the scenario in which ‘silesaurids’ are an outgroup to Dinosauria and highlight the differences between these hypotheses for the evolution of the ornithischian axial skeleton (figure 6B).

**Figure 6.**
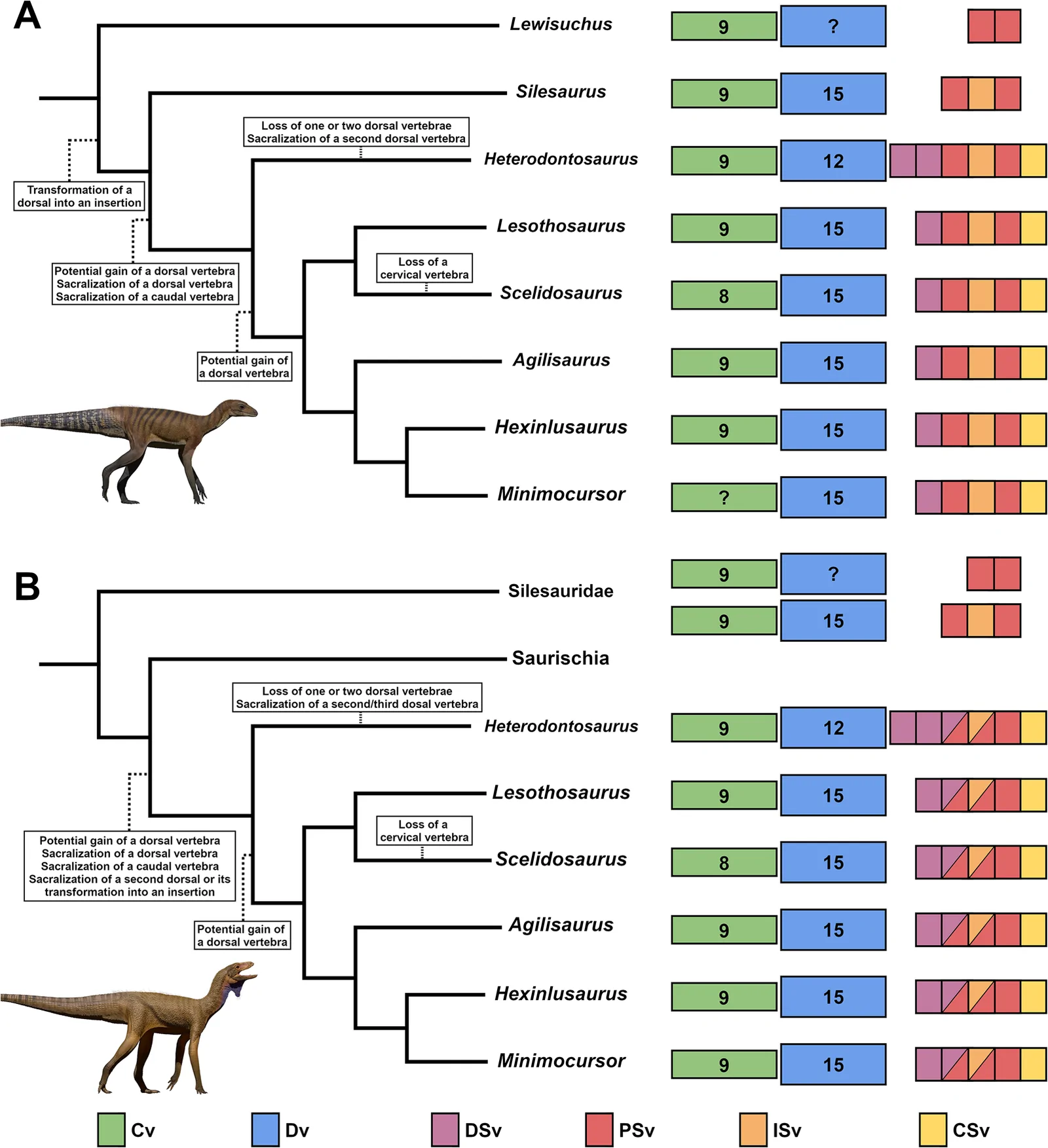
Evolution of the precaudal axial skeleton of early-branching ornithischians. (A) Scenario considering ‘silesaurids’ to represent a paraphyletic series of early ornithischians. (B) Scenario considering ‘silesaurids’ to be an outgroup to Dinosauria. Artistic reconstructions by Matheus Fernandes Gadelha. Abbreviations: Cv, cervical vertebrae; CSv, caudosacral vertebra; Dv, dorsal vertebrae; DSv, dorsosacral vertebrae; ISv, inserted sacral vertebra; PSv, primordial sacral vertebrae.

Following the interpretation that ‘silesaurids’ present nine cervical vertebrae, the cervical series is not subject to change across most of the sampled taxa (figure 6). The exception is *Scelidosaurus*, which only has eight cervical vertebrae. Considering its 24th presacral vertebra to represent a dorsosacral, *Scelidosaurus* would match most other saphornithischians in having 15 dorsal vertebrae and 5 sacral vertebrae. The loss of one cervical did not affect the remaining precaudal regions, which maintained their previous composition, leading us to conclude that a vertebra was lost in this taxon. The incompleteness of the precaudal series in other non-eurypodan thyreophorans precludes identification if this is exclusive to *Scelidosaurus*, or more widespread across related taxa.

The earliest diverging ornithischians have the ancestral condition of a sacrum exclusively composed of the primordial sacrals (e.g. *Asilisaurus* and *Lewisuchus* [53,84]), as observed in most non-dinosaurian archosauromorphs and herrerasaurians. More derived ‘silesaurids’ bear an additional sacral vertebra located between their primordial sacrals (e.g. *Gondwanax* and *Silesaurus* [19,20]). As discussed below, this vertebra most probably represents a posterior dorsal that was incorporated into the sacrum. Even in light of ‘silesaurids’ as the ancestral ornithischians, there is still a gap of knowledge between the three-vertebra sacrum of these ‘silesaurids’ and the five-vertebrae sacrum of saphornithischians. *Pisanosaurus* is the single taxon that might bear four sacral vertebrae; unfortunately, its sacral count has been the subject of debate across multiple studies (see discussion in [116–118]). Regardless of its sacral count, the material is poorly informative, being preserved as a mould in rock [116]. It is not possible to confidently identify the origin of the additional pair of putative sacral elements. It is noteworthy that the last dorsal is fused into the sacrum of some specimens of *Silesaurus* (e.g. ZPAL Ab III/404/3 [20,82]). Although this vertebra is not part of the sacrum, it might suggest that a dorsosacral vertebra was the fourth sacral added to the sacrum of ornithischians. Support for this hypothesis is weak, and it is therefore regarded as preliminary and contingent on the acquisition of additional data.

The sacrum of saphornithischians bears five vertebrae, as observed in early-diverging members (e.g. *Eocursor*, *Lesothosaurus* [32,33,52]), thyreophorans (e.g. *Scutellosaurus* and perhaps *Scelidosaurus* [64,119]) and neornithischians (e.g. *Agilisaurus*, *Hexinlusaurus*, *Minimocursor*, [44,50,58]). This condition is most often interpreted to represent one dorsosacral and four ‘true sacrals’, that is, sacral vertebrae bearing individual sacral ribs [32–34]. A subsequent transformation is observed in the sacrum of heterodontosaurids, which incorporated another dorsal as a second dorsosacral ([21,32,34,49,52, 120,121]). Despite the additional dorsosacral, the anatomy of the heterodontosaurid sacrum is similar to that of other early-branching saphornithischians, with the second to sixth sacrals of heterodontosaurids corresponding to the first to fifth sacrals of other saphornithischians [34,120]. The origin of the second, third and fourth sacrals is not clear, although we consider these to respectively represent the first primordial sacral, an inserted sacral and the second primordial sacral. Their identities will be further discussed in their respective dedicated topics.

The evolution of the dorsal series is the least straightforward. Among non-saphornithischians, only *Silesaurus* preserves a complete dorsal series, comprising 15 vertebrae [65,82]. Non-heterodontosaurid saphornithischians share with *Silesaurus* the presence of 15 dorsal vertebrae. Despite the shared dorsal number, one unambiguous dorsosacral has been incorporated into the saphornithischian sacrum [32–34]; therefore, there was an increase in the total presacral count of saphornithischians compared with *Silesaurus* (26 vs. 25, see TPC in table 1), and one additional vertebra is present in the dorsal series of most saphornithischians. The dorsal series of heterodontosaurids appears to be the most modified, with *Heterodontosaurus* bearing only 12 dorsal vertebrae, and the 6-vertebrae sacrum of *Manidens*, suggesting a similar reduction in dorsal count ([21,49,121]). One vertebra was unambiguously incorporated into the sacrum as a second dorsosacral element, whereas the fate of the remaining vertebrae is less certain. Their incorporation into either the cervical series or the sacrum is unlikely, as *Heterodontosaurus* retains the plesiomorphic count of nine cervical vertebrae and its sacrum includes only one additional element relative to other saphornithischians. Therefore, it is probable that at least one dorsal vertebra was lost in heterodontosaurids (see reduced total presacral count for this taxon, TPC in table 1), but it is harder to discern if a second loss occurred. The sum of the dorsal, dorsosacral and inserted vertebrae suggests that *Silesaurus* has 16 vertebrae that originate from the trunk. Considering either the second sacral of saphornithischians to represent a dorsosacral or the third sacral to be an insertion, *Heterodontosaurus* would have 15 and other saphornithischians would have 17 vertebrae with their origins in the trunk (see §4 on the origins of inserted sacrals). Compared with the ancestral condition in early saurischians and *Silesaurus*, it is certain that one trunk vertebra was lost in *Heterodontosaurus* and one vertebra was acquired in more derived saphornithischians. Evaluation of the total presacral count of these taxa confirms the occurrence of somitogenesis in Saphornithischia. Whereas saurischians and ‘silesaurids’ have a total presacral count of 25 vertebrae, *Heterodontosaurus* shows a decrease to 24 vertebrae, showing that one vertebra was lost, whereas other saphornithischians such as *Agilisaurus* and *Lesothosaurus* show an increase to 26 vertebrae and provide evidence that one vertebra was formed. It is not possible to determine whether somitogenesis occurred only in saphornithischians closer to Genasauria, with a single vertebra subsequently lost in *Heterodontosaurus*, or whether an additional element was already present in the ancestral saphornithischian condition, followed by the loss of two vertebrae in *Heterodontosaurus*. A parsimonious interpretation favours the former scenario, as it requires fewer transformational steps; however, additional fossil material will be necessary to adequately test and corroborate either hypothesis.

As presented earlier, the hypothesis that ‘silesaurids’ are the sister taxon to Dinosauria does not fundamentally change the observations here discussed (figure 6B). Because a cervical count of nine vertebrae is shared among early dinosauromorphs (e.g. *Lagosuchus*), ‘silesaurids’ (e.g. *Lewisuchus*), core ornithischians (e.g. *Lesothosaurus*) and saurischians (e.g. *Buriolestes*, *Gnathovorax*), the exclusion of ‘silesaurids’ from Ornithischia would not alter the ancestral condition for the latter clade. Thus, the count of eight cervical vertebrae in *Scelidosaurus* would remain unique and provide evidence that this taxon lost one cervical element. The plesiomorphic condition of the sacrum would therefore remain unchanged, with a sacrum composed of only the two primordial vertebrae occurring in most early archosaurs and the earliest diverging saurischians (e.g. *Gnathovorax*, *Tawa*), suggesting that this was the most probably ancestral condition for Ornithischia. There is agreement that the first, fourth and fifth sacrals of core ornithischians respectively represent a dorsosacral, the second primordial sacral and a caudosacral vertebra ([20,32], this contribution), but the identity of the second and third vertebrae is less certain. We use the condition of ‘silesaurids’ and their similarity to core ornithischians to argue that the second vertebra represents the first primordial sacral and the third vertebra represents an insertion, but if ‘silesaurids’ are not ornithischians, this argument loses support. As will be further discussed below, it is hard to identify the primordial sacrals in saphornithischians. Without the ‘silesaurid’ condition to act as a guide, it remains ambiguous if these vertebrae represent a dorsosacral and primordial (as in [32]), or a primordial and an insertion (as in [20]). Independent of which hypothesis is supported, dorsosacral and inserted vertebrae seem to stem from the dorsal series, and therefore the total presacral count would remain unchanged. Owing to this stability in the total presacral count, the interpretations of the dorsal series are also similar independent of the ‘silesaurid’ affinities, albeit still showing evidence of a complex scenario. The reduced dorsal count of heterodontosaurids still suggests at least one dorsal vertebra was lost, whereas genasaurians show evidence that one additional element was acquired, and therefore supporting further meristic changes to the early ornithischian axial skeleton. In summary, the discussion on the cervical and dorsal evolution of ornithischians remains equal or near unaltered if ‘silesaurids’ are not part of the clade, with the greatest change regarding sacral homology. Without ‘silesaurids’ to suggest the third sacral of saphornithischians corresponds to an insertion, it becomes ambiguous if the first primordial sacral of saphornithischians is represented by their second or third sacrals.

It is clear that the evolution of the axial series in early ornithischians is more complex than in early saurischians. The ancestral ornithischian most probably matched the total presacral count observed in saurischian dinosaurs, with 9 cervicals, 16 dorsals and 2 primordial sacrals. More derived members successively incorporated vertebrae from the trunk and tail into their sacra, originating dorsosacrals/insertions and caudosacral, respectively. There is strong evidence that additional vertebrae were acquired and pre-existing vertebrae were lost in the precaudal series of early ornithischians (changes in the total presacral count). Thus, unlike saurischians, the axial evolution of the earliest ornithischians cannot be accounted for solely by changes in Hox gene expression and homeotic incorporation, supporting the involvement of meristic events early in ornithischian evolution.

### 4.3. The origins of the inserted sacral, a possible variation of the dorsosacral condition

The sacrum of stem-archosaurs is primarily composed of two sacral vertebrae, and various lineages independently modified this number, increasing in sacral complexity [20,122]. Sacra are primarily modified by incorporating vertebrae from the trunk (dorsosacral vertebrae), which are added anterior to the first primordial sacral, or vertebrae from the tail (caudosacral vertebrae), which are added posterior to the second primordial sacral (e.g. [20,28,29]). These were considered the only options by most studies until a new option was proposed in Nesbitt [20], an inserted sacral. In the framework developed by the author, primordial sacral vertebrae consistently attach near key positions in the ilium: the first primordial sacral attaching near the base of the preacetabular process and the pubic peduncle, and the second primordial sacral attaching near the base of the postacetabular process and the ischiatic peduncle of the ilium. The remaining sacral vertebrae would be identified in relation to the position of the primordial sacrals: vertebrae anterior to the first primordial are dorsosacrals, vertebrae posterior to the second primordial are caudosacrals and vertebrae in between the primordials are insertions [20]. Following this methodology, inserted sacrals would be present in some pseudoschians (e.g. *Batrachotomus* and multiple poposauroids) and several dinosaur groups: ‘silesaurids’ (e.g. *Gondwanax*, *Silesaurus*), core ornithischians (e.g. *Heterodontosaurus*, *Lesothosaurus*), sauropodomorphs (e.g. *Massospondylus*) and neotheropods (e.g. *Allosaurus*, *Carnotaurus*, *Coelophysis,* and *Dilophosaurus*).

Whereas Nesbitt [20] discussed the distinction of the inserted sacral from dorsosacral and caudosacral vertebrae based on its position between the primordial sacrals, the origin of the inserted sacral remained uncertain. It remains unclear whether vertebrae positioned between the primordial sacrals must originate within the sacral series (i.e. a meristic origin) or whether they can be incorporated from the dorsal or caudal series (i.e. a homeotic origin). If the first hypothesis is correct and the inserted sacral occurs via somitogenesis, we should expect that the presacral and caudal series would maintain a consistent number of vertebrae despite the appearance of the inserted sacral. If the second hypothesis is correct and the inserted sacral originates through incorporation from an adjacent region, a corresponding loss of one vertebral element should be expected either in the presacral or in the caudal series. Evaluation of the available sample supports the second hypothesis, as the appearance of an inserted sacral vertebra is consistently and strongly correlated with the loss of a dorsal vertebra. *Silesaurus* has 15 dorsal vertebrae, one less than the possible plesimorphic condition observed in saurischians, and it bears an inserted sacral. The presacral series of sauropodomorphs that bear an inserted sacral (*Adeopapposaurus*, *Massospondylus* and *Sarahsaurus*) closely approaches the count of closely related species that bear a dososacral (e.g. *Lufengosaurus*, *Xingxiulong*, *Yizhousaurus*), with the most significant differences being the disappearance of the dorsosacral and appearance of the inserted sacral. Although we are not certain that coelophysoids bear an inserted sacral, if such a condition were present, the appearance of the putative ‘inserted’ vertebrae in these taxa would also be associated with the loss of a dorsal vertebra. Although this pattern may be coincidental, the consistent association between the loss of a dorsal vertebra and the appearance of an inserted sacral suggests that this relationship is unlikely to be so. Furthermore, the insertions observed in ornithischians, sauropodomorphs and potentially theropods were acquired convergently. Yet, they follow the same pattern of dorsal vertebral loss associated with sacral gain, supporting the interpretation that this pattern is significant and not a coincidence restricted to a single lineage. Albeit based on a limited sample, the inserted sacral of dinosaurs is tentatively associated with the loss of one dorsal vertebra, supporting that this condition is derived from an incorporation of a dorsal vertebra into the sacrum.

Proposing that the inserted sacral is derived from a dorsal vertebra raises the question if it should continue to be treated as a distinct condition or if it should just be considered homologous and ‘synonymous’ to the dorsosacral condition. We argue that despite sharing an origin within the trunk, these two conditions should be treated separately, or at least be different stages of a transformation series. This distinctiveness is justified by the same principle Nesbitt [20] used to differentiate insertions from the remaining conditions; the distinct positions relative to the primordial sacrals suggest that these vertebrae were incorporated into the sacrum via distinct pathways. Standard dorsosacral vertebrae seem to be incorporated by changes in Hox gene expression leading to the manifestation of sacral-like characteristics in the last dorsal vertebrae and functionally becoming a part of the sacrum. Dorsosacrals could be understood as trunk vertebrae attaching anterior to the first primordial sacral, with their anatomy becoming more sacral-like, in a similar process that gives rise to caudosacral vertebrae and cervicalized ‘dorsal’ vertebrae. When insertions are present, a vertebra originating from the dorsal series becomes positioned between the pair of primordial sacrals. We tentatively suggest that inserted sacrals may originate from an anterior shift in sacral Hox gene expression, which causes the first primordial sacral to be formed by one vertebra anteriorly. Under this scenario, a vertebral element becomes effectively ‘trapped’ between the two primordial sacrals, resulting in an insertion. Inserted sacrals could therefore be interpreted as arising from an anterior shift of the first primordial sacral into the dorsal series, with the interposed vertebra subsequently undergoing sacralization. These distinct morphologies, and potentially distinct developmental trajectories, suggest that, despite a probable origin from dorsal vertebrae, inserted sacrals are sufficiently different from dorsosacrals to warrant treatment as a separate sacralization phenomenon.

Despite this interesting perspective on the origins of the inserted sacral, it should be noted that this is a preliminary proposal based on the currently available sample of informative early dinosaur axial skeletons. Among dinosaurs with well-preserved precaudal series, only a few show evidence of an inserted condition. Moreover, the axial evolution of pseudosuchians also requires further investigation to determine whether their inserted sacral was acquired through a pathway similar to that of dinosaurs. Additionally, the axial evolution of the earliest pterosauromorphs remains to be thoroughly explored. A lack of adequate models to test the feasibility of the hypothesis here presented hampers interpreting the axial evolution of early dinosaurs. Despite being dinosaurs, the axial skeleton of birds is highly modified and might not allow us to test the idea of the first primordial sacral advancing anteriorly into the dorsal series. It might be possible to test it in crocodilians, which retain a plesiomorphic sacral anatomy [3,122,123]. Although none of the extant crocodilians naturally develop a third sacral, developmental biology studies show that it is possible to cause a sacralization event in mouse and chick embryos (e.g. [11]), casting optimistic light on its feasibility. Nevertheless, there are still challenges in identifying the primordial sacrals in archosaurs with complex sacra, which will impact the interpretation of the origin of the remaining sacrals.

### 4.4. Challenges in identifying the primordial sacrals in taxa with multiple sacral vertebrae

As discussed in detail above, the ancestral archosauromorph sacrum is composed of two sacral vertebrae, termed the primordial sacrals [20,29,83). In taxa that retain few sacral vertebrae, it is simple to identify the primordial vertebrae, but it becomes challenging to identify them once many new elements are incorporated into the sacrum [20]. In taxa approaching the plesiomorphic condition, the primordial sacrals retain their defining anatomy, and evaluation of either rib morphology or the corresponding scars on the medial surface of the ilium allows reliable identification of the primordial sacrals [20]. As different lineages increase in sacral complexity, additional vertebrae occupy the iliac articulation, causing the primordial sacrals to lose their characteristic anatomy and thereby hindering their identification [20]. A solution to this challenge was proposed by Nesbitt [20]; according to the author, the position of the primordial sacrals remains consistent in early archosaurs. The first primordial sacral is consistently located in the anterior portion of the ilium, with it attaching the base of the preacetabular process and pubic peduncle. The second primordial sacral is consistently located in the posterior portion of the ilium, with its anterior portion located near/at the ischiatic peduncle of the ilium and its posterior portion being located at the base of the postacetabular process. Based on this consistent positioning of the primordial sacrals in early croc-line and bird-line archosaurs, even in taxa with three or more sacral vertebrae, Nesbitt [20] proposed using this position-based model to identify their primordial sacrals (in addition to other informative morphologies like centrum and rib morphology). This model has been widely used to aid in identifying sacral vertebral homologies across early archosaurs (e.g. [19,30,45,62,101,102,122, 124–126]) and has also been applied to more derived groups (e.g. [127]). Despite the wide use of this model, there are still challenges in identifying primordial sacrals in early dinosaurs. This mainly manifests in the difficulty of identifying the first primordial sacral and whether a third sacral component is an insertion or a dorsosacral.

The potential polymorphism observed in *Saturnalia* illustrates this issue. The scars in the ilium of the holotype (MCP 3844-PV) closely match positions predicted by Nesbitt's model, whereas in the paratype (MCP 3845-PV), the presence of a dorsosacral vertebra complicates this comparison. The dorsosacral vertebra of the paratype attaches to the ilium near the base of the preacetabular process and the pubic peduncle [30], a position expected for the first primordial sacral, although it cannot be homologized with that vertebra. Interestingly, in this specimen, the modified sacral rib fused to the transverse process is only present on the right side, whereas on the left side, the rib is absent (or unfused and subsequently lost), and the transverse process is slightly shorter and does not seem to contact the medial surface of the ilium. Comparisons between the size and morphology of the following sacral and those of the holotype confirm that the second sacral of MCP 3845-PV is the first primordial sacral. Whereas the morphology securely confirms this is not the first primordial sacral, if we were to exclusively consider the location of its sacral scar, it could lead to an incorrect identification that this is the first primordial sacral. The multiple specimens and the primordial sacrals retaining the plesiomorphic morphology help in solving the case in *Saturnalia*. This is not true for most other dinosaurs, as observed in *Xingxiulong*, a more derived sauropodomorph. This taxon is considered to have a dorsosacral vertebra, which is described to articulate above the base of the pubic peduncle, whereas the primordial sacral is described to articulate slightly anterior to the base of the ischiatic peduncle [128]. When considering positional relationships alone, the dorsosacral could be interpreted as the first primordial sacral, with the original primordial sacral effectively representing an insertion. However, the anatomy of the first vertebra suggests affinities with the dorsal series, which would support a dorsosacral identification [128]. Therefore, exclusively relying on the attachment site on the ilium could lead to a misidentification of vertebral homology. A dorsosacral articulating near the contact between the preacetabular process and pubic peduncle appears to be common in sauropodomorphs (*Bagualia*, *Leonerasaurus*, *Mussaurus*, *Patagosaurus*, *Saturnalia*, *Xingxiulong* [21,30,109,127,128]), further disagreeing with the position only-based model. The position of the dorsosacral in sauropodomorphs presents a disagreement with the position-based model of Nesbitt [20], and it suggests that other ribs can articulate in the position suggested to belong to the first primordial sacral. Therefore, caution is important when interpreting homology of sacral elements based on the location of their sacral rib scars. Fortunately, the large record of early-branching sauropodomorphs with a simpler sacral composition helps in correctly identifying their homology and better understanding the evolution of the sauropod sacrum.

In contrast to sauropodomorphs, theropods exhibit a more complex sacral anatomy and a smaller number of taxa with a simple sacrum, making identification of the first primordial sacral more challenging. Nesbitt [20] interpreted/scored *Coelophysis* and *Dilophosaurus* as having inserted sacrals, in addition to one dorsosacral and one caudosacral. Based on these scorings, we can conclude that the author interpreted the second and fourth vertebrae to be the first and second primordial sacrals, respectively. This homology was accepted and followed in some studies (e.g. [87]), although others disagreed and considered the first two sacrals of coelophysoids to be dorsosacrals and the third sacral vertebra to be the first primordial sacral [54]. The sacrum of *Dilophosaurus* was redescribed by Marsh & Rowe [45] as lacking any inserted sacral, with only four sacrals identified of which the second and third were primordials based on rib shape. Similarly, in *Coelophysis*, only sacrals 3 and 4 bear true sacral ribs homologous with the primordial sacrals (first-hand examination of TMP 1984.063.0037, TMP 1984.063.0038 and TMP 1984.063.0039). Therefore, despite having a sacral count of five, the sacrum is composed of two dorsosacrals, the two primordial sacrals and one caudosacral. From the available specimens of early theropods, no inserted sacrals can be confidently identified based on rib anatomy, suggesting that iliac articulation alone cannot be used in the identification of primordial sacral vertebrae.

Within Ornithischia, given the near absence of taxa with a simple sacral anatomy and the fact that all saphornithischians possess a complex sacrum composed of five or more vertebrae, ornithischians further contribute to the challenges already discussed for saurischian dinosaurs. In addition, there have been few attempts to understand sacral homology in ornithischians, most of which were undertaken in studies with a broader focus on early dinosaurs/archosaurs [20,29], and such analyses are rarely included in descriptions of individual ornithischian taxa. Among the ornithischians sampled in this study, the only explicit attempt to identify sacral homology concerns SAM-PK-K1107, now referred to as *Lesothosaurus*, in which the third and fourth sacrals were interpreted as the primordial ones [32]. This interpretation predates the recognition that an inserted condition could occur [20] and was based on the assumption that the primordial sacrals must remain adjacent to one another. Although SAM-PK-K1107 was treated at the time as a distinct taxon (*Stormbergia*), Nesbitt [20], based on other specimens, considered *Lesothosaurus* to possess an inserted sacral vertebra.

The complex arrangement of five sacral vertebrae, the derived rib morphology and the disputed origin of ornithischians hamper robust assessment of sacral vertebral homology. Under the hypothesis that ‘silesaurids’ are a series of early-branching ornithischians, we suggest that the second sacral vertebra of saphornithischians represents the first primordial vertebra, and the third sacral vertebra would correspond to an insertion. The proposed arrangement would match the condition observed in ‘silesaurids’ and fit the position based on the model by Nesbitt [20], with the first primordial sacral articulating near the pubic peduncle. Furthermore, this is corroborated by the identification of the first ‘true sacral’ corresponding to the first primordial sacral of *Protoceratops*, whose primordial rib articulates in the pubic peduncle [15].

Despite the high anatomical disparity achieved by ornithischians, nearly all early-diverging taxa retain only three sacral vertebrae articulating in the main iliac body (region between the pubic and ischiatic peduncle) and positions roughly homologous to those seen in ‘silesaurids’ (figure 7). This not only supports the homology of the ‘silesaurid’ condition but also shows that the attachment site of the second and third sacrals remains consistent across early-diverging ornithischians, independent of their identity (dorsosacral and primordial vs. primordial and insertion). Despite the proposed homology, we are aware that the identity of these two vertebrae is probably to remain ambiguous as far as the origin of ornithischians remains debated. Solving this problem probably requires further field efforts and new well-preserved specimens that fill our gaps in knowledge, and a more thorough re-evaluation of the sacral anatomy across saphornithischians, which will help to test this hypothesis.

**Figure 7.**
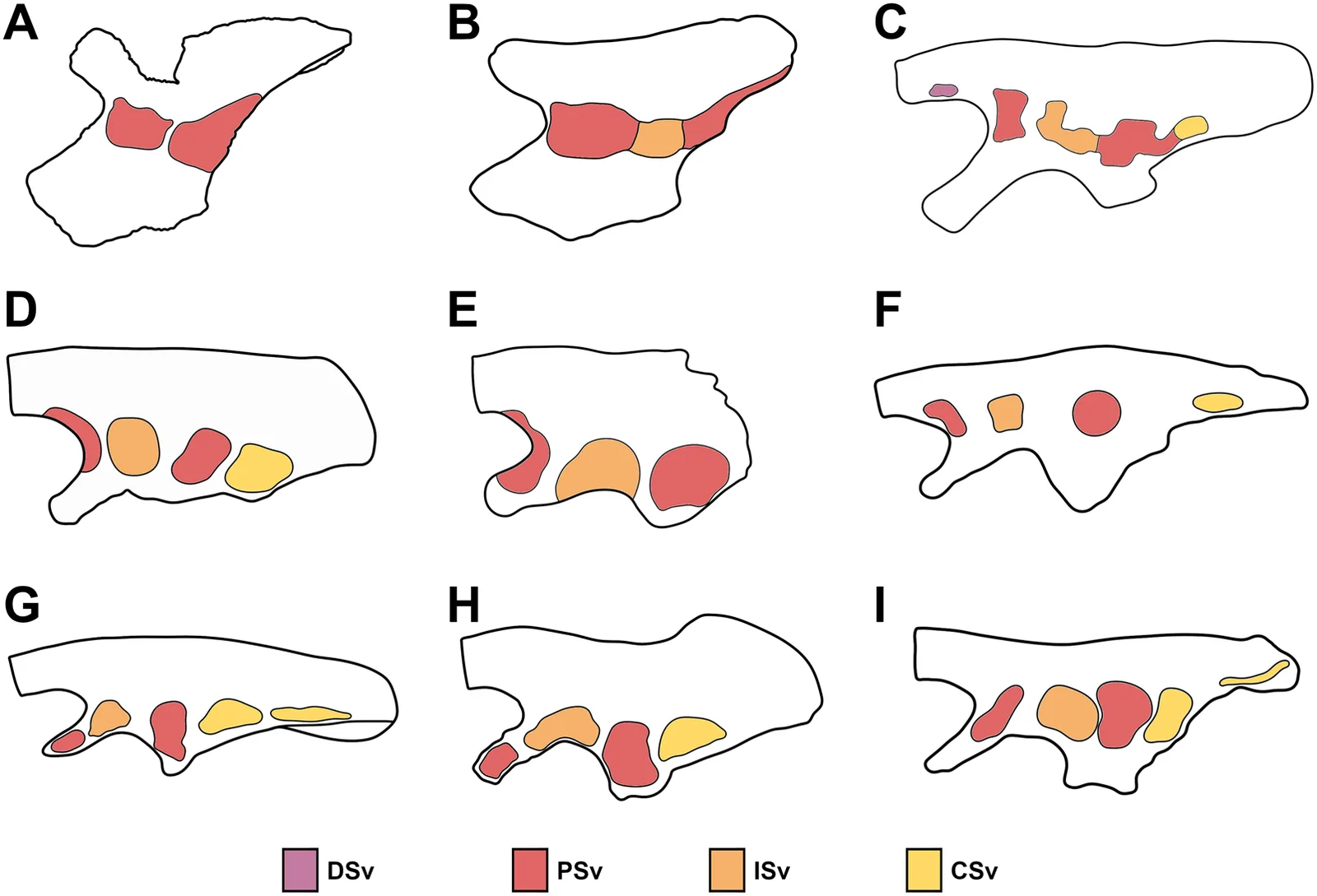
Schematic showing the approximate positions of the sacral scars in the medial surface of the ilium in early-branching ornithischians. Preacetabular processes removed for spatial reasons. (A) *Asilisaurus* (NMT RB159 [84]); (B) *Silesaurus* (ZPAL AbIII/362 [72]); (C) *Lesothosaurus* (SAM-PK-K1107 [32]); (D) *Scelidosaurus* (NHMUK R6704 [64]); (E) *Hexinlusaurus* (ZDM T6002 [50]); (F) *Yinlong* (IVPP V18682 [129]); (G) *Hypsilophodon* (NHMUK VP R196 [130]); (H) *Tenontosaurus* (AMNH 3040 [131]); (I) *Dysalotosaurus* (HMN dy II of [132], now MB.R.1718, see [133]). Abbreviations: Cv, cervical vertebrae; CSv, caudosacral vertebra; dv, dorsal vertebrae; DSv, dorsosacral vertebrae; ISv, inserted sacral vertebra; PSv, primordial sacral vertebrae.

Although the position of the first primordial sacral is more complex and does not always correspond to that proposed by Nesbitt [20] in the specimens examined here, the second primordial sacral consistently occupies the position identified by that author, near the ischiadic peduncle and postacetabular process, in all evaluated taxa. This recognition is relevant because it allows all vertebrae posterior to this point to be identified as caudosacral vertebrae, thereby establishing homology and improving our understanding of the axial evolution in early dinosaurs.

### 4.5. Perspective for future research

Whereas sauropodomorphs are represented by a proportionately large sample, the records of ornithischians and theropods are less extensive. This limited representation of theropods and ornithischians complicates interpretations of dinosaur axial evolution, and additional well-preserved specimens are needed to test the hypotheses presented here. Fortunately, recent fieldwork has yielded new materials with well-preserved axial series. The revision of axial counts and zonations presented here will probably aid future studies in interpreting taxa known from incomplete axial series and in the earliest stages of dinosaur evolution.

The identification of primordial sacrals will probably remain problematic in derived taxa, where these elements lose their characteristic anatomy. Understanding the evolution of complex sacra will thus benefit from extensive sampling of earlier branching species with well-preserved material, underscoring the importance of ongoing fieldwork. Although beyond our expertise, developmental studies on sacral variability in living archosaurs would be welcome and could help test whether the evolution of an inserted sacral follows the dorsal-series pathway proposed here.

## 5. Conclusions

Changes to the axial zonation occurred early and convergently across different dinosaurian lineages. Saurischians retain a relatively stable number of vertebrae derived from the presacral region, and all instances of cervicalization and sacralization involve vertebrae incorporated from the dorsal or caudal series. Ornithischians, by contrast, exhibit a more complex evolutionary pattern, reflecting both their debated ancestry and their more extensively modified sacrum. Although the earliest ornithischians possessed only two sacral vertebrae, a third sacral was soon acquired and inserted between the primordials. Comparisons between this inserted sacral and the sacrum of saphornithischians suggest that this condition persists in more derived members of the clade. There is unambiguous evidence for the loss of presacral vertebrae in saphornithischians, along with probable evidence for vertebral acquisition. Unlike early saurischians, whose axial evolution is dominated by homeotic changes, ornithischian evolution appears to reflect a combination of homeotic and meristic processes. In dinosaurs, the presence of an inserted sacral is consistently associated with the loss of a dorsal vertebra, indicating that inserted sacrals derive from the dorsal series rather than representing a case of somitogenesis. Although identifying primordial sacrals in the taxa with complex sacra remains challenging, the second primordial sacral appears to consistently occupy a conserved position, facilitating the recognition of sacral homology.

## Data Availability

The data used in this contribution are the axial counts of the selected early-branching dinosaurs. The vertebral numbers and studies used to justify those are made available in tables 1 and 2. All evaluated material corresponds to specimens deposited in scientific collections, listed here or at their respective references, and available for further study.

## Glossary

AMNH,: American Museum of Natural History, New York, USA
BP,: Evolutionary Studies Institute, University of the Witwatersrand, Johannesburg, South Africa
CAMSM,: Sedgwick Museum, University of Cambridge, Cambridge
CAPPA/UFSM,: Centro de Apoio à Pesquisa Paleontológica da Quarta Colônia da Universidade Federal de Santa Maria, São João do Polêsine, Brazil
IVPP: Institute of Vertebrate Paleontology and Paleoanthropology, Beijing, China
MB,: Museum Für Naturkunde, Berlin, Germany
MCP,: Museu de Ciências e Tecnologia, Pontifícia Universidade Católica do Rio Grande do Sul, Porto Alegre, Brazil
NHMUK,: Natural History Museum, London British Museum, London, UK
NMT,: National Museum of Tanzania, Dar es Salaam, Tanzania
PVSJ,: Instituto y Museo de Ciencias Naturales, Universidad Nacional de San Juan, San Juan, Argentina
SAM,: South African Museum (Iziko Museums of Cape Town), Cape Town, South Africa
TMM,: Vertebrate Paleontology Laboratory, University of Texas at Austin, Austin, Texas
TMP,: Royal Tyrrell Museum of Palaeontology, Drumheller, Canada
ZDM,: Zigong Dinosaur Museum, Dashanpu, People's Republic of China
ZPAL,: Institute of Paleobiology, Polish Academy of Sciences, Warsaw, Poland.

## References

[1] Aires AS , Reichert LM, Müller RT, Pinheiro FL, Andrade MB. 2021 Development and evolution of the notarium in Pterosauria. J. Anat. 238, 400–415. (doi:10.1111/joa.13319) PMC7812132 33026119

[2] Jones KE , Gonzalez S, Angielczyk KD, Pierce SE. 2020 Regionalization of the axial skeleton predates functional adaptation in the forerunners of mammals. Nat. Ecol. Evol. 4, 470–478. (doi:10.7910/DVN/YMUHLU) 32015524

[3] Müller J , Scheyer TM, Head JJ, Barrett PM, Werneburg I, Ericson PG, Pol D, Sánchez-Villagra MR. 2010 Homeotic effects, somitogenesis and the evolution of vertebral numbers in recent and fossil amniotes. Proc. Natl Acad. Sci. U. S. A. 107, 2118–2123. (doi:10.1073/pnas.0912622107) PMC2836685 20080660

[4] Roberts LE , Head JJ. 2026 Evolution of the reptile spine reveals independent trajectories to axial skeletal complexity in amniotes. Nat. Commun. 17, 5304. (doi:10.1038/s41467-026-72071-x) PMC13269487 41991554

[5] Romer AS . 1956 Osteology of the reptiles. Chicago, IL: University of Chicago Press.

[6] Figueirido B , Serrano FJ, Pérez-Ramos A, Esteban JM, Ferrón HG, Martín-Serra A. 2022 Body-axis organization in tetrapods: a model-system to disentangle the developmental origins of convergent evolution in deep time. Biol. Lett. 18, 20 220 047. (doi:10.1098/rsbl.2022.0047) PMC8984341 35382583

[7] Gomez C , Özbudak EM, Wunderlich J, Baumann D, Lewis J, Pourquié O. 2008 Control of segment number in vertebrate embryos. Nature 454, 335–339. (doi:10.1038/nature07020) 18563087

[8] Burke AC , Nelson CE, Morgan BA, Tabin C. 1995 Hox genes and the evolution of vertebrate axial morphology. Development 121, 333–346. (doi:10.1242/dev.121.2.333) 7768176

[9] Casaca A , Santos AC, Mallo M. 2014 Controlling Hox gene expression and activity to build the vertebrate axial skeleton. Dev. Dyn. 243, 24–36. (doi:10.1002/dvdy.24007) 23813547

[10] Gaunt SJ . 1994 Conservation in the Hox code during morphological evolution. Int. J. Dev. Biol. 38, 549–552. (doi:10.1387/ijdb.7848839) 7848839

[11] Gérard M , Zákány J, Duboule D. 1997 Interspecies exchange of a Hoxd enhancer *in vivo* induces premature transcription and anterior shift of the sacrum. Dev. Biol. 190, 32–40. (doi:10.1006/dbio.1997.8679) 9331329

[12] Richardson MK , Allen SP, Wright GM, Raynaud A, Hanken J. 1998 Somite number and vertebrate evolution. Development 125, 151–160. (doi:10.1242/dev.125.2.151) 9486789

[13] Zákány J , Gérard M, Favier B, Duboule D. 1997 Deletion of a HoxD enhancer induces transcriptional heterochrony leading to transposition of the sacrum. EMBO J. 16, 4393–4402. (doi:10.1093/emboj/16.14.4393) PMC1170065 9250683

[14] Carpenter K , DiCroce T, Kinneer B, Simon R. 2013 Pelvis of *Gargoyleosaurus* (Dinosauria: Ankylosauria) and the origin and evolution of the ankylosaur pelvis. PLoS ONE 8, e79887. (doi:10.1371/journal.pone.0079887) PMC3828194 24244573

[15] Tereshchenko VS . 2021 Axial skeleton of subadult *protoceratops andrewsi* from Djadokhta Formation (Upper Cretaceous, Mongolia). Paleontol. J. 55, 1408–1457. (doi:10.1134/S0031030121120030)

[16] Wilson JA , Upchurch P. 2009 Redescription and reassessment of the phylogenetic affinities of *Euhelopus zdanskyi* (Dinosauria: Sauropoda) from the Early Cretaceous of China. J. Syst. Paleontol. 7, 199–239. (doi:10.1017/S1477201908002691)

[17] Wu WH , Zhou CF, Oliver W, Sekiya T, Dong ZM. 2013 A new gigantic sauropod dinosaur from the Middle Jurassic of Shanshan, Xinjiang. Glob. Geol. 32, 437–446.

[18] Moro D , Kerber L, Müller RT, Pretto FA. 2021 Sacral co-ossification in dinosaurs: the oldest record of fused sacral vertebrae in Dinosauria and the diversity of sacral co-ossification patterns in the group. J. Anat. 238, 828–844. (doi:10.1111/joa.13356) PMC7930772 33164207

[19] Müller RT . 2025 A new “silesaurid” from the oldest dinosauromorph-bearing beds of South America provides insights into the early evolution of bird-line archosaurs. Gondwana Res. 137, 13–28. (doi:10.1016/j.gr.2024.09.007)

[20] Nesbitt SJ . 2011 The early evolution of archosaurs: relationships and the origin of major clades. Bull. Am. Mus. Nat. Hist. 352, 1–292. (doi:10.1206/352.1)

[21] Pol D , Garrido A, Cerda IA. 2011 A new sauropodomorph dinosaur from the Early Jurassic of Patagonia and the origin and evolution of the sauropod-type sacrum. PLoS ONE 6, e14572. (doi:10.1371/journal.pone.0014572) PMC3027623 21298087

[22] Owen R . 2022 Report on British fossil reptiles. Part II. Rep. Br. Assoc. Adv. Sci. 11, 60–204. (doi:10.4324/9780429355653-42)

[23] Schaeffer J . 2024 Osteological redescription of the holotype of *Plateosaurus trossingensis* (Dinosauria: Sauropodomorpha) from the Upper Triassic of SW Germany and its phylogenetic implications. J. Syst. Paleontol. 22, 2 335 387. (doi:10.1080/14772019.2024.2335387)

[24] Colbert EH . 1989 The Triassic dinosaur *Coelophysis*. Mus. North Ariz. Bull. 57, 1–160.

[25] Rozadilla S , Agnolín FL, Novas FE. 2019 Osteology of the Patagonian ornithopod *Talenkauen santacrucensis* (Dinosauria, Ornithischia). J. Syst. Paleontol. 17, 2043–2089. (doi:10.1080/14772019.2019.1582562)

[26] Müller RT , Langer MC, Bronzati M, Pacheco CP, Cabreira SF, Dias-da-Silva S. 2018 Early evolution of sauropodomorphs: anatomy and phylogenetic relationships of a remarkably well-preserved dinosaur from the Upper Triassic of southern Brazil. Zool. J. Linnean Soc. 184, 1187–1248. (doi:10.1093/zoolinnean/zly009)

[27] Novas FE . 1994 New information on the systematics and postcranial skeleton of *Herrerasaurus ischigualastensis* (Theropoda: Herrerasauridae) from the Ischigualasto Formation (Upper Triassic) of Argentina. J. Vertebr. Paleontol. 13, 400–423. (doi:10.1080/02724634.1994.10011523)

[28] Sereno PC . 1999 The evolution of dinosaurs. Science 284, 2137–2147. (doi:10.1126/science.284.5423.2137) 10381873

[29] Langer MC , Benton MJ. 2006 Early dinosaurs: a phylogenetic study. J. Syst. Paleontol. 4, 309–358. (doi:10.1017/S1477201906001970)

[30] Marsola JCA , Bittencourt JS, Butler RJ, Da Rosa ÁtS, Sayão JM, Langer MC. 2018 A new dinosaur with theropod affinities from the Late Triassic Santa Maria Formation, South Brazil. J. Vertebr. Paleontol. 38, e1531878. (doi:10.1080/02724634.2018.1531878)

[31] Wilson JA . 2011 Anatomical terminology for the sacrum of sauropod dinosaurs. Contrib. Mus. Paleontol. Univ. Michigan 32, 59–69.

[32] Butler RJ . 2005 The ‘fabrosaurid’ ornithischian dinosaurs of the Upper Elliot Formation (Lower Jurassic) of South Africa and Lesotho. Zool. J. Linnean Soc. 145, 175–218. (doi:10.1111/j.1096-3642.2005.00182.x)

[33] Butler RJ . 2010 The anatomy of the basal ornithischian dinosaur *Eocursor parvus* from the Lower Elliot Formation (Late Triassic) of South Africa. Zool. J. Linnean Soc. 160, 648–684. (doi:10.1111/j.1096-3642.2009.00631.x)

[34] Sereno PC . 2012 Taxonomy, morphology, masticatory function and phylogeny of heterodontosaurid dinosaurs. ZooKeys 226, 1–225. (doi:10.3897/zookeys.223.2840) PMC3491919 23166462

[35] Müller RT , Garcia MS. 2020 A paraphyletic ‘Silesauridae’ as an alternative hypothesis for the initial radiation of ornithischian dinosaurs. Biol. Lett. 16, 20 200 417. (doi:10.1098/rsbl.2020.0417) PMC7480155 32842895

[36] Ezcurra MD , Garcia MS, Novas FE, Müller RT, Agnolín FL, Chatterjee S. 2025 A new herrerasaurian dinosaur from the Upper Triassic Upper Maleri Formation of southcentral India. R. Soc. Open Sci. 12, 1–20. (doi:10.1098/rsos.250081) PMC12077243 40370605

[37] Garcia MS , Müller RT. 2025 Triassic pterosaur precursors of Brazil: catalog, evolutionary context, and a new hypothesis for phylogenetic relationships of pterosauromorpha. Anais Acad. Brasil. Ciências 97, e20240844. (doi:10.1590/0001-3765202520240844) 40008775

[38] Fonseca AO , Reid IJ, Venner A, Duncan RJ, Garcia MS, Müller RT. 2024 A comprehensive phylogenetic analysis on early ornithischian evolution. J. Syst. Paleontol. 22, 2346577. (doi:10.1080/14772019.2024.2346577)

[39] Martínez RN , Colombi, CE, Ezcurra, MD, Abelín, DO, Cerda I, Alcober OA. 2025 A Carnian theropod with unexpectedly derived features during the first dinosaur radiation. Nat. Ecol. Evol. 9, 2238–2247. (doi:10.1038/s41559-025-02868-4) 41087647

[40] Srivastava S , Nesbitt SJ. 2026 A new taxon of saurischian dinosaur from the *Coelophysis* Quarry of New Mexico, USA (Triassic: latest Norian or Rhaetian) highlights herrerasaurian diversity in the latest Triassic. Pap. Palaeontol. 12, e70069. (doi:10.1002/spp2.70069)

[41] Zahner M , Brinkmann W. 2019 A Triassic averostran-line theropod from Switzerland and the early evolution of dinosaurs. Nat. Ecol. Evol. 3, 1146–1152. (doi:10.1038/s41559-019-0941-z) PMC6669044 31285577

[42] Fonseca AO , Bem FP, Dalle-Laste VZ, Garcia MS, Müller RT. 2026 Osteology of the axial skeleton of *Macrocollum itaquii* (Dinosauria: Sauropodomorpha) and its implications for early sauropodomorph cervicalization. Zool. J. Linnean Soc. 206, zlag034. (doi:10.1093/zoolinnean/zlag034)

[43] Martínez RN . 2009 *Adeopapposaurus mognai*, gen. Et sp. nov. (Dinosauria: Sauropodomorpha), with comments on adaptations of basal sauropodomorpha. J. Vertebr. Paleontol. 29, 142–164. (doi:10.1671/039.029.0102)

[44] Peng G . 1992 Jurassic ornithopod *Agilisaurus louderbacki* (Ornithopoda: Fabrosauridae) from Zigong, Sichuan, China. Vertebr. PalAsiat. 30, 39.

[45] Marsh AD , Rowe TB. 2020 A comprehensive anatomical and phylogenetic evaluation of *Dilophosaurus wetherilli* (Dinosauria, Theropoda) with descriptions of new specimens from the Kayenta Formation of northern Arizona. J. Paleontol. 94, 1–103. (doi:10.1017/jpa.2020.14)

[46] Galton PM . 1973 On the anatomy and relationships of *Efraasia diagnostica* (Huene) n. gen., a prosauropod dinosaur (Reptilia: Saurischia) from the Upper Triassic of Germany. Paläontol. Z. 47, 229–255. (doi:10.1007/BF02985709)

[47] Sereno PC , Martínez RN, Alcober OA. 2013 Osteology of *Eoraptor lunensis* (Dinosauria, Sauropodomorpha). J. Vertebr. Paleontol. 32, 83–179. (doi:10.1080/02724634.2013.820113)

[48] Pacheco C , Müller RT, Langer M, Pretto FA, Kerber L, Dias da Silva S. 2019 *Gnathovorax cabreirai*: a new early dinosaur and the origin and initial radiation of predatory dinosaurs. PeerJ 7, e7963. (doi:10.7717/peerj.7963) PMC6844243 31720108

[49] Galton PM . 2014 Notes on the postcranial anatomy of the heterodontosaurid dinosaur *Heterodontosaurus tucki*, a basal ornithischian from the Lower Jurassic of South Africa. Rev. Paléobiol. 33, 97–141.

[50] He XL , Cai K. 1984 The Middle Jurassic dinosaurian fauna from Dashanpu, Zigong, Sichuan. Vol 1, the ornithopod dinosaurs. Chengdu, China: Sichuan Scientific and Technological Publishing House.

[51] Knoll F . 2010 A primitive sauropodomorph from the Upper Elliot Formation of Lesotho. Geol. Mag. 147, 814–829. (doi:10.1017/S001675681000018X)

[52] Baron MG , Norman DB, Barrett PM. 2017 Postcranial anatomy of *Lesothosaurus diagnosticus* (Dinosauria: Ornithischia) from the Lower Jurassic of southern Africa: implications for basal ornithischian taxonomy and systematics. Zool. J. Linnean Soc. 179, 125–168. (doi:10.1111/zoj.12434)

[53] Agnolín FL , Egil FB, Ezcurra MD, Langer MC, Novas FE. 2022 New specimens provide insights into the anatomy of the dinosauriform *Lewisuchus admixtus* Romer, 1972 from the Upper Triassic levels of the Chañares Formation, NW Argentina. Anat. Rec. 305, 1119–1146. (doi:10.1002/ar.24731) 34358415

[54] Martínez RN , Apaldetti C. 2017 A Late Norian—Rhaetian coelophysid Neotheropod (Dinosauria, Saurischia) from the Quebrada Del Barro Formation, northwestern Argentina. Ameghiniana 54, 488–505. (doi:10.5710/AMGH.09.04.2017.3065)

[55] Young CC . 1941 A complete osteology of *Lufengosaurus heunei* young (gen. et sp. nov.) from Lufeng, Yunnan, China. Palaeontol. Sin. Ser. C 7, 1–53.

[56] Müller RT , Langer MC, Dias-da-Silva S. 2018 An exceptionally preserved association of complete dinosaur skeletons reveals the oldest long-necked sauropodomorphs. Biol. Lett. 14, 20 180 633. (doi:10.1098/rsbl.2018.0633) PMC6283919 30463923

[57] Barrett PM , Chapelle KE, Staunton CK, Botha J, Choiniere JN. 2019 Postcranial osteology of the neotype specimen of *Massospondylus carinatus* Owen, 1854 (Dinosauria: Sauropodomorpha) from the Upper Elliot Formation of South Africa. Palaeontol. Afr. 53, 114–178. (https://hdl.handle.net/10539/26829)

[58] Manitkoon S , Deesri U, Khalloufi B, Nonsrirach T, Suteethorn V, Chanthasit P, Boonla W, Buffetaut E. 2023 A new basal neornithischian dinosaur from the Phu Kradung Formation (Upper Jurassic) of northeastern Thailand. Diversity 15, 851. (doi:10.3390/d15070851)

[59] Chapelle KE , Barrett PM, Botha J, Choiniere JN. 2019 *Ngwevu intloko*: a new early sauropodomorph dinosaur from the Lower Jurassic Elliot Formation of South Africa and comments on cranial ontogeny in *Massospondylus carinatus*. PeerJ 7, e7240. (doi:10.7717/peerj.7240) PMC6687053 31403001

[60] You HL , Azuma Y, Wang T, Wang YM, Dong ZM. 2014 The first well-preserved coelophysoid theropod dinosaur from Asia. Zootaxa, 3873, 233–249. (doi:10.11646/zootaxa.3873.3.3) 25544219

[61] Alcober OA , Martínez RN. 2010 A new herrerasaurid (Dinosauria, Saurischia) from the Upper Triassic Ischigualasto Formation of northwestern Argentina. ZooKeys 63, 55. (doi:10.3897/zookeys.63.550) PMC3088398 21594020

[62] Marsh AD , Rowe TB. 2018 Anatomy and systematics of the sauropodomorph *Sarahsaurus aurifontanalis* from the Early Jurassic Kayenta Formation. PLoS ONE 13, e0204007. (doi:10.1371/journal.pone.0204007) PMC6179219 30304035

[63] Langer MC . 2003 The pelvic and hind limb anatomy of the stem-sauropodomorph *Saturnalia tupiniquim* (Late Triassic, Brazil). PaleoBios 23, 1–30.

[64] Norman DB . 2020 *Scelidosaurus harrisonii* from the Early Jurassic of Dorset, England: postcranial skeleton. Zool. J. Linnean Soc. 189, 47–157. (doi:10.1093/zoolinnean/zlz078)

[65] Piechowski R , Dzik J. 2010 The axial skeleton of *Silesaurus opolensis*. J. Vertebr. Paleontol. 30, 1127–1141. (doi:10.1080/02724634.2010.483547)

[66] Novas FE , Agnolin FL, Ezcurra MD, Temp Müller R, Martinelli AG, Langer MC. 2021 Review of the fossil record of early dinosaurs from South America, and its phylogenetic implications. J. South Am. Earth Sci. 110, 103 341. (doi:10.1016/j.jsames.2021.103341)

[67] Wang YM , You HL, Wang T. 2017 A new basal sauropodiform dinosaur from the Lower Jurassic of Yunnan Province, China. Sci. Rep. 7, 41 881. (doi:10.1038/srep41881) PMC5312170 28205592

[68] Zhang QN , You HL, Wang T, Chatterjee S. 2018 A new sauropodiform dinosaur with a ‘sauropodan’ skull from the Lower Jurassic Lufeng Formation of Yunnan Province, China. Sci. Rep. 8, 13 464. (doi:10.1038/s41598-018-31874-9) PMC6128897 30194381

[69] Hu S . 1993 A short report on the occurrence of *Dilophosaurus* from Jinning County, Yunnan Province. Vertebr. PalAsiat. 31, 65–69. (doi:10.1017/s0016756800028211)

[70] Tykoski RS . 1998 The osteology of Syntarsus kayentakatae and its implications for ceratosaurid phylogeny, PhD thesis, University of Texas at Austin, Austin, TX.

[71] Lü J , Li T, Zhong S, Azuma Y, Fujita M, Dong Z, Ji Q. 2007 New yunnanosaurid dinosaur (Dinosauria, Prosauropoda) from the Middle Jurassic Zhanghe Formation of Yuanmou, Yunnan Province of China. Mem. Fuk. Prefect. Dinosaur Mus. 6, 1–15.

[72] Piechowski R , Tałanda M. 2020 The locomotor musculature and posture of the early dinosauriform *Silesaurus opolensis* provides a new look into the evolution of Dinosauromorpha. J. Anat. 236, 1044–1100. (doi:10.1111/joa.13155) PMC7219628 32003023

[73] Spielmann JA , Lucas SG, Rinehart LF, Hunt AP, Heckert AB, Sullivan, RM. 2007 Oldest records of the Late Triassic theropod dinosaur *Coelophysis bauri*. New Mexico Mus. Nat. Hist. Sci. Bull. 41, 384–401.

[74] Raath MA . 1977 The anatomy of the Triassic theropod Syntarsus rhodesiensis (Saurischia: Podokesauridae) and a consideration of its biology. Unpublished PhD thesis, Rhodes University, Grahamstown.

[75] Martinez RN , Sereno PC, Alcober OA, Colombi CE, Renne PR, Montañez IP, Currie BS. 2011 A basal dinosaur from the dawn of the dinosaur era in southwestern Pangaea. Science 331, 206–210. (doi:10.1126/science.1198467) 21233386

[76] Sereno PC , Forster CA, Rogers RR, Monetta AM. 1993 Primitive dinosaur skeleton from Argentina and the early evolution of Dinosauria. Nature, 361, 64–66. (doi:10.1038/361064a0)

[77] Sereno PC , Novas FE. 1994 The skull and neck of the basal theropod *Herrerasaurus ischigualastensis*. J. Vertebr. Paleontol. 13, 451–476. (doi:10.1080/02724634.1994.10011525)

[78] Garcia MS . 2023 Gnathovorax cabreirai, um dinossauro excepcionalmente bem preservado do Triássico superior do Sul do Brasil. Unpublished MSc thesis, Universidade Federal de Santa Maria, Santa Maria.

[79] Nesbitt SJ , Smith ND, Irmis RB, Turner AH, Downs A, Norell MA. 2009 A complete skeleton of a Late Triassic saurischian and the early evolution of dinosaurs. Science 326, 1530–1533. (doi:10.1126/science.1180350) 20007898

[80] Bittencourt JS , Arcucci AB, Marsicano CA, Langer MC. 2015 Osteology of the Middle Triassic archosaur *Lewisuchus admixtus* Romer (Chañares Formation, Argentina), its inclusivity, and relationships amongst early dinosauromorphs. J. Syst. Paleontol. 13, 189–219. (doi:10.1080/14772019.2013.878758)

[81] Hugi JC . 2008 The axial and posterior appendicular morphology of the first theropod skeleton (Saurischia, Dinosauria) of Switzerland (Late Triassic; frick, Canton Aargau). PhD thesis, Bibliothèque cantonale jurassienne, Switzerland.

[82] Dzik, J . 2003 A beaked herbivorous archosaur with dinosaur affinities from the Early Late Triassic of Poland. J. Vertebr. Paleontol. 23, 556–574. (doi:10.1671/A1097)

[83] Ezcurra MD . 2016 The phylogenetic relationships of basal archosauromorphs, with an emphasis on the systematics of proterosuchian archosauriforms. PeerJ 4, e1778. (doi:10.7717/peerj.1778) PMC4860341 27162705

[84] Nesbitt SJ , Langer MC, Ezcurra MD. 2020 The anatomy of *Asilisaurus kongwe*, a dinosauriform from the Lifua Member of the Manda Beds (∼ Middle Triassic) of Africa. Anat. Rec. 303, 813–873. (doi:10.1002/ar.24287) 31797580

[85] Bittencourt JDS , Kellner AWA. 2009 The anatomy and phylogenetic position of the Triassic dinosaur *Staurikosaurus pricei* Colbert, 1970. Zootaxa 2079, 1–56. (doi:10.11646/zootaxa.2079.1.1)

[86] Galton PM . 2000 Are *Spondylosoma* and *Staurikosaurus* (Santa Maria Formation, Middle-Upper Triassic, Brazil) the oldest saurischian dinosaurs? PalZ 74, 393–423. (doi:10.1007/BF02988109)

[87] Spiekman SN , Ezcurra MD, Butler RJ, Fraser NC, Maidment SC. 2021 *Pendraig milnerae*, a new small-sized coelophysoid theropod from the Late Triassic of Wales. R. Soc. Open Sci. 8, 210 915. (doi:10.1098/rsos.210915) PMC8493203 34754500

[88] Fonseca AO , Bem FP, Müller RT. 2025 Osteology of the appendicular skeleton of *Macrocollum itaquii* (Dinosauria: Sauropodomorpha) sheds light on early dinosaur wrist evolution. Zool. J. Linnean Soc. 205, zlaf100. (doi:10.1093/zoolinnean/zlaf100)

[89] McPhee BW , Bonnan MF, Yates AM, Neveling J, Choiniere JN. 2015 A new basal sauropod from the pre-Toarcian Jurassic of South Africa: evidence of niche-partitioning at the sauropodomorph–sauropod boundary? Sci. Rep. 5, 13 224. (doi:10.1038/srep13224) PMC4541066 26288028

[90] Yates AM . 2007 The first complete skull of the Triassic dinosaur *Melanorosaurus* Haughton (Sauropodomorpha: Anchisauria). Spec. Pap. Palaeontol. 77, 9–55.

[91] Nesbitt SJ , Flynn JJ, Pritchard AC, Parrish JM, Ranivoharimanana L, Wyss AR. 2015 Postcranial osteology of *Azendohsaurus madagaskarensis* (? Middle to Upper Triassic, Isalo Group, Madagascar) and its systematic position among stem archosaur reptiles. Bull. Am. Mus. Nat. Hist. 398, 1–126. (doi:10.1206/amnb-899-00-1-126.1)

[92] Rytel A , Böhmer C, Spiekman SN, Tałanda M. 2024 Extreme neck elongation evolved despite strong developmental constraints in bizarre Triassic reptiles—implications for neck modularity in archosaurs. R. Soc. Open Sci. 11, 240 233. (doi:10.1098/rsos.240233) PMC11285776 39076823

[93] Trotteyn, MJ, Ezcurra MD. 2014 Osteology of *Pseudochampsa ischigualastensis* gen. Et comb. nov. (Archosauriformes: Proterochampsidae) from the Early Late Triassic Ischigualasto Formation of northwestern Argentina. PLoS ONE 9, e111388. (doi:10.1371/journal.pone.0111388) PMC4245112 25426846

[94] Dilkes D , Sues HD. 2009 Redescription and phylogenetic relationships of Doswellia kaltenbachi (Diapsida: Archosauriformes) from the Upper Triassic of Virginia. J. Vertebr. Paleontol. 29, 58–79. (doi:10.1080/02724634.2009.10010362)

[95] Sen K . 2005 A new rauisuchian archosaur from the Middle Triassic of India. Palaeontology 48, 185–196. (doi:10.1111/j.1475-4983.2004.00438.x)

[96] Agnolin FL , Ezcurra MD. 2019 The validity of *Lagosuchus talampayensis* Romer, 1971 (Archosauria, Dinosauriformes), from the Late Triassic of Argentina. Breviora 565, 1–21. (doi:10.3099/0006-9698-565.1.1)

[97] SJ Nesbitt *et al.* 2017 The earliest bird-line archosaurs and the assembly of the dinosaur body plan. Nature 544, 484–487. (doi:10.1038/nature22037) 28405026

[98] Sereno PC , Arcucci AB. 1994 Dinosaurian precursors from the Middle Triassic of Argentina: *Lagerpeton chanarensis*. J. Vertebr. Paleontol. 13, 385–399. (doi:10.1080/02724634.1994.10011522)

[99] Sereno PC , Arcucci AB. 1994 Dinosaurian precursors from the Middle Triassic of Argentina: *Marasuchus lilloensis*, gen. nov. J. Vertebr. Paleontol. 14, 53–73. (doi:10.1080/02724634.1994.10011538)

[100] SF Cabreira *et al.* 2016 A unique Late Triassic dinosauromorph assemblage reveals dinosaur ancestral anatomy and diet. Curr. Biol. 26, 3090–3095. (doi:10.1016/j.cub.2016.09.040) 27839975

[101] Nesbitt SJ , Patellos E, Kammerer CF, Ranivoharimanana L, Wyss AR, Flynn JJ. 2023 The earliest-diverging avemetatarsalian: a new osteoderm-bearing taxon from the Triassic (? Earliest Late Triassic) of Madagascar and the composition of avemetatarsalian assemblages prior to the radiation of dinosaurs. Zool. J. Linnean Soc. 199, 327–353. (doi:10.1093/zoolinnean/zlad038)

[102] Nesbitt SJ , Barrett PM, Werning S, Sidor CA, Charig AJ. 2013 The oldest dinosaur? A Middle Triassic dinosauriform from Tanzania. Biol. Lett. 9, 20 120 949. (doi:10.1098/rsbl.2012.0949) PMC3565515 23221875

[103] Marsh AD , Parker WG, Langer MC, Nesbitt SJ. 2019 Redescription of the holotype specimen of *Chindesaurus bryansmalli* Long and Murry, 1995 (Dinosauria, Theropoda), from Petrified Forest National Park, Arizona. J. Vertebr. Paleontol. 39, e1645682. (doi:10.1080/02724634.2019.1645682)

[104] Brochu CA . 2003 Osteology of *Tyrannosaurus rex*: insights from a nearly complete skeleton and high-resolution computed tomographic analysis of the skull. J. Vertebr. Paleontol. 22, 1–138. (doi:10.1080/02724634.2003.10010947)

[105] Zhao XJ , Benso RBJ, Brusatte SL, Currie PJ. 2010 The postcranial skeleton of *Monolophosaurus jiangi* (Dinosauria: Theropoda) from the Middle Jurassic of Xinjiang, China, and a review of Middle Jurassic Chinese theropods. Geol. Mag. 147, 13–27. (doi:10.1017/S0016756809990240)

[106] Griffin CT , Wynd BM, Munyikwa D, Broderick TJ, Zondo M, Tolan S, Langer MC, Nesbitt SJ, Taruvinga HR. 2022 Africa's oldest dinosaurs reveal early suppression of dinosaur distribution. Nature 609, 313–319. (doi:10.1038/s41586-022-05133-x) 36045297

[107] Chen XY , Wang YM, Zhang QN, Wang T, You HL. 2025 A new species of *Xingxiulong* (Dinosauria, Sauropodomorpha) from the Lower Jurassic Lufeng Formation of Yunnan Province, China. Hist. Biol. 37, 1–10. (doi:10.1080/08912963.2025.2458130)

[108] Galton PM , Van Heerden J, Yates AM. 2005Postcranial anatomy of referred specimens of the sauropodomorph dinosaur *Melanorosaurus* from the Upper Triassic of South Africa. In Thunder-lizards: the sauropodomorph dinosaurs (eds V Tidwell, K Carpenter), pp. 1–37. Bloomington, IN: Indiana University Press.

[109] Otero A , Pol D. 2013 Postcranial anatomy and phylogenetic relationships of Mussaurus patagonicus (Dinosauria, Sauropodomorpha). J. Vertebr. Paleontol. 33, 1138–1168. (doi:10:1080/02724634.2013769444)

[110] Galton PM . 1976 Prosauropod dinosaurs (Reptilia: Saurischia) of North America. Postilla 169, 1–98. (doi:10.1016/s0016-6995(85)80065-6)

[111] Cau A . 2024 A unified framework for predatory dinosaur macroevolution. Boll. Soc. Paleontol. Ital. 63, 1–19. (doi:10.4435/BSPI.2024.08)

[112] Müller RT , Garcia MS. 2023 A new silesaurid from Carnian beds of Brazil fills a gap in the radiation of avian line archosaurs. Sci. Rep. 13, 4981. (doi:10.1038/s41598-023-32057-x) PMC10090097 37041170

[113] Norman DB , Baron MG, Garcia MS, Müller RT. 2022 Taxonomic, palaeobiological and evolutionary implications of a phylogenetic hypothesis for Ornithischia (Archosauria: Dinosauria). Zool. J. Linnean Soc. 196, 1273–1309. (doi:10.1093/zoolinnean/zlac062)

[114] Müller RT , Ezcurra MD, Garcia MS, Agnolín FL, Stocker MR, Novas FE, Soares MB, Kellner AWA, Nesbitt SJ. 2023 New reptile shows dinosaurs and pterosaurs evolved among diverse precursors. Nature 620, 589–594. (doi:10.1038/s41586-023-06359-z) 37587301

[115] MD Ezcurra *et al.* 2020 Enigmatic dinosaur precursors bridge the gap to the origin of Pterosauria. Nature 588, 445–449. (doi:10.1038/s41586-020-3011-4) 33299179

[116] Agnolín FL , Rozadilla S. 2018 Phylogenetic reassessment of *Pisanosaurus mertii* Casamiquela, 1967, a basal dinosauriform from the Late Triassic of Argentina. J. Syst. Paleontol. 16, 853–879. (doi:10.1080/14772019.2017.1352623)

[117] Baron MG . 2019 *Pisanosaurus mertii* and the Triassic ornithischian crisis: could phylogeny offer a solution? Hist. Biol. 31, 967–981. (doi:10.1080/08912963.2017.1410705)

[118] JB Desojo *et al.* 2020 The Late Triassic Ischigualasto Formation at Cerro Las Lajas (La Rioja, Argentina): fossil tetrapods, high-resolution chronostratigraphy, and faunal correlations. Sci. Rep. 10, 12 782. (doi:10.1038/s41598-020-67854-1) PMC7391656 32728077

[119] Breeden BT , Raven TJ, Butler RJ, Rowe TB, Maidment SCR. 2021 The anatomy and palaeobiology of the early armoured dinosaur *Scutellosaurus lawleri* (Ornithischia: Thyreophora) from the Kayenta Formation (Lower Jurassic) of Arizona. R. Soc. Open Sci. 8, 201 676. (doi:10.1098/rsos.201676) PMC8292774 34295511

[120] Butler RJ , Porro LB, Galton PM, Chiappe LM. 2012 Anatomy and cranial functional morphology of the small-bodied dinosaur Fruitadens haagarorum from the Upper Jurassic of the USA. PLoS ONE 7, e31556. (doi:10.1371/journal.pone.0031556) PMC3324477 22509242

[121] Becerra MG , Pol D, Cerda IA, Porro LB, Rauhut OW. 2025 Postcranial osteology of Manidens condorensis (Ornithischia: Heterodontosauridae) from the Lower Jurassic of Argentina: 3D reconstruction, histology, and phylogenetic implications. Papers in Palaeontology, 11, e70053. (doi:10.1002/spp2.70053)

[122] Griffin CT , Stefanic CM, Parker WG, Hungerbühler A, Stocker MR. 2017 Sacral anatomy of the phytosaur *Smilosuchus adamanensis*, with implications for pelvic girdle evolution among Archosauriformes. J. Anat. 231, 886–905. (doi:10.1111/joa.12681) PMC5696133 28836268

[123] Mansfield JH , Abzhanov A. 2010 Hox expression in the American alligator and evolution of archosaurian axial patterning. J. Exp. Zool. B Mol. Dev. Evol. 314B, 629–644. (doi:10.1002/jez.b.21364) 20623505

[124] LePore CN , McLain MA. 2024 Variation in the sacrum of phytosaurs: new evidence from a partial skeleton of *Machaeroprosopus mccauleyi*. J. Anat. 244, 959–976. (doi:10.1111/joa.14007) PMC11095306 38284134

[125] Li H , Xing X, Jiang J, Liu J, Brusatte SL, Bi S. 2025 New material of a non-averostran neotheropod dinosaur from the Lower Jurassic Lufeng Formation of Yunnan, south-western China. Zool. J. Linnean Soc. 204, zlaf034. (doi:10.1093/zoolinnean/zlaf034)

[126] Schachner ER , Irmis RB, Huttenlocker AK, Sanders K, Cieri RL, Fox M, Nesbitt SJ. 2020 Osteology of the late Triassic bipedal archosaur *Poposaurus gracilis* (Archosauria: Pseudosuchia) from western North America. Anat. Rec. 303, 874–917. (doi:10.1002/ar.24298) 31814308

[127] Gomez KL , Carballido JL, Pol D. 2021 The axial skeleton of *Bagualia alba* (Dinosauria: Eusauropoda) from the Early Jurassic of Patagonia. Palaeontol. Electron. 24, a37. (doi:10.26879/1176) 39887763

[128] Wang YM , Wang T, Yang ZW, You HL. 2020 Cranium and vertebral column of *Xingxiulong cheng**i* (Dinosauria: Sauropodomorpha) from the Early Jurassic of China. Anat. Rec. 303, 772–789. (doi:10.1002/ar.24305) 31804026

[129] Han F , Forster Ca, Xu X, Clark JM. 2018 Postcranial anatomy of Yinlong downsi (Dinosauria: Ceratopsia) from the Upper Jurassic Shishugou Formation of China and the phylogeny of basal ornithischians. J. Syst. Palaeontol. 16, 1159–1187. (doi:10.1080/14772019.2017.1369185)

[130] Galton PM . 1974 The ornithischian dinosaur *Hypsilophodon* from the Wealden of the Isle of Wight. Bull. Br. Mus. 25, 1–152. (doi:10.5962/p.313819)

[131] Forster CA . 1990 The postcranial skeleton of the ornithopod dinosaur *Tenontosaurus tilletti*. J. Vertebr. Paleontol. 10, 273–294. (doi:10.1080/02724634.1990.10011815)

[132] Galton Pm . 1981 Dryosaurus, a hypsilophodontid dinosaur from the Upper Jurassic of North America and Africa postcranial skeleton. Paläontologische Zeitschrift, 55, 271–312. (doi:10.1007/BF02988144)

[133] Hübner T . 2018 The postcranial ontogeny of *Dysalotosaurus lettowvorbecki* (Ornithischia: Iguanodontia) and implications for the evolution of ornithopod dinosaurs. Palaeontogr. Abteil. A Palaozool. 310, 43–120. (doi:10.1127/pala/2018/0072)

